# ‘It’s not just about the dinner; it’s about everything else that we do’: A qualitative study exploring how Meals on Wheels meet the needs of self‐isolating adults during COVID‐19

**DOI:** 10.1111/hsc.13634

**Published:** 2021-11-12

**Authors:** Angeliki Papadaki, Becky Ali, Ailsa Cameron, Miranda E. G. Armstrong, Paul Isaacs, Kali S. Thomas, Emily A. Gadbois, Paul Willis

**Affiliations:** ^1^ Centre for Exercise, Nutrition and Health Sciences School for Policy Studies University of Bristol Bristol UK; ^2^ Centre for Research in Health and Social Care School for Policy Studies University of Bristol Bristol UK; ^3^ U.S. Department of Veterans Affairs Medical Center Providence Rhode Island USA; ^4^ Brown University School of Public Health Providence Rhode Island USA

**Keywords:** community meals, COVID‐19, home‐delivered meals, lockdown, meals on wheels, older adults, qualitative research

## Abstract

Meals on Wheels (MoWs), a service offered by local authorities in England, deliver meals to older, housebound and/or vulnerable adults, who might otherwise not be able to acquire and prepare their own meals. Research suggests that MoWs provide benefits beyond nutrition. Little is known about the actual interactions between service providers and clients, particularly during the COVID‐19 pandemic. The aim of this small‐scale, formative study was to explore MoWs service providers’ experiences and their perceptions around the benefits and challenges faced by the service, and understand how these experiences changed during the first UK national lockdown. Semi‐structured interviews were conducted in September 2020 with 18 service providers of MoWs (drivers who deliver the meals, service coordinators and managers) in two local authorities in England, and analysed thematically. Participants indicated that benefits of the service encompassed those to clients (e.g. welfare checks, encouraging independence and identifying and addressing isolation and loneliness), employees (e.g. sense of pride, rewarding relationships with clients) and the wider community (e.g. reducing pressures on families), and described MoWs as the ‘fourth emergency service’ (e.g. being the first responders to emergency situations). Participants identified several challenges faced by the MoWs service, including organisational challenges (e.g. funding cuts and closures, lack of appropriate publicity to raise awareness of the service) and restrictions on time spent with clients. The pandemic and lockdown resulted in increased demand on resources, concerns about client and staff wellbeing and uncertainty about how the service will cope if lockdowns continue. These findings provide important insights regarding the wide benefits of MoWs and the challenges the service faces, which can be used as the formative research base to guide future interventions and policies to protect vulnerable adults, not only during the COVID‐19 pandemic, but beyond.

**What is known about this topic?**

- Meals on Wheels (MoWs) provide meals to vulnerable individuals who might not be able to prepare or acquire their own meals.
- MoWs provide wider benefits in addition to improving nutritional intakes.
- No study has examined the staff‐client interactions, benefits and challenges faced by MoWs in England, or how experiences with service provision changed during the COVID‐19 pandemic.

**What does this paper add?**

- MoWs offer multiple benefits to clients, employees and the wider community.
- MoWs experience many challenges, such as funding cuts and closures, lack of appropriate publicity and reduced time spent with clients.
- Further challenges introduced by the pandemic included an increased demand on resources and concerns about client and staff wellbeing and how the service will cope if lockdowns continue.

## 1 INTRODUCTION

In March 2020, an estimated 1.5 million people most vulnerable to COVID‐19 were instructed to shield in England. A further 17.7 million adults aged ≥70 years were instructed to stay at home as much as possible, as they were considered to be at increased risk of infection (UK Cabinet Office, 2020). Many of these individuals might not have had sufficient support from families and community resources to access adequate and nutritious food, nor the financial resources or capability to prepare their own meals. These factors are established as contributing to undernutrition in older adults who are housebound (Locher et al., 2008). Coupled with the fact that approximately one in seven adults aged ≥65 years is at medium or high risk of malnutrition (BAPEN, 2018), the need for all vulnerable individuals to access nutritious food during the pandemic became imperative.

Meals on Wheels (MoWs), as a service offered by 42% of local authorities across the United Kingdom (UK) (National Association of Care Catering, 2018), might be crucial in delivering food to housebound adults. Consistent evidence suggests that MoWs improve nutrient intakes in older adults (Wright et al., 2015; Zhu & An, 2013) and offer benefits that extend beyond nutrition, including the provision of welfare checks, opportunities for social interaction, decreased rates of isolation and improved quality of life (Campbell et al., 2015; Thomas et al., 2016). The use of MoWs has also been linked to decreased need for residential care, by helping vulnerable individuals remain independent in their homes and communities and ‘age in place’ (Altshuler & Schimmel, 2010; Thomas & Mor, 2013). These beneficial outcomes present essential preventative measures, and would undoubtedly have become more important during the pandemic, which increased demand for the service in the UK (Sustain, 2020).

A recent qualitative study in the United States of America (USA) that explored the perceptions of MoWs drivers about their interactions with clients confirmed many of the aforementioned benefits (Thomas et al., 2020). Much of the research on the benefits of MoWs, however, comes from the USA and Canada (Winterton et al., 2013), and therefore might not be generalisable to the UK. No research to date has examined the perceptions of MoWs service providers in England, particularly with regards to the benefits of MoWs and the challenges faced by the service. Further, no study has explored how these experiences changed during the first national UK lockdown (March‐June 2020). The aim of this study was therefore to explore these issues among drivers who deliver the meals, service coordinators and managers in two local authorities (LAs) in South‐West England. The study was funded as part of an initiative to provide evidence to inform policy actions for protecting the most vulnerable (Papadaki et al., 2021), particularly during the pandemic. Findings from this small‐scale, local study contribute to the evidence base informing future interventions and policies aiming to enhance the MoWs service.

## 2 METHODS

### 2.1 Study design and participants

Semi‐structured interviews were conducted in September 2020 with employees of MoWs services in two LAs in South‐West England, which were conveniently and purposively selected according to geographic location (urban and semi‐urban). MoWs managers in the two LAs were invited by email to act as gatekeepers for participant recruitment. This involved circulating a study invitation to all MoWs employees who engage directly with clients (*n* = 23 and *n* = 20, in the LAs serving urban only and semi‐urban areas, respectively). A total of 19 employees initially expressed interest in participating. Of these, one was not available for an interview; the remaining 18 employees participated. Data collection and analysis proceeded in parallel and saturation was deemed to have been reached after the sixteenth interview (Harris et al., 2009). The study is reported following the Consolidated Criteria for Reporting Qualitative Research (COREQ) guidelines (Tong et al., 2007) (Table S1).

### 2.2 Data collection

Semi‐structured interviews, lasting 28–56 min, were conducted via telephone and audio recorded. Two interview guides were developed to explore the experiences of MoWs drivers, and employees with office‐based duties (Appendix S1‐S2). The guides were not piloted, but were informed by recent research exploring the interactions between MoWs drivers and clients in the USA (Thomas et al., 2020), and developed further to explore topics that addressed the specific objectives of the current research. In summary, the interviews explored participants’ experiences working within the service and delivering meals to clients, their perceptions on the benefits of MoWs to older adults, and the challenges they faced in their job and those faced by the service more generally. Participants were also asked to describe a typical meal delivery interaction and to discuss whether they offer any other assistance to their clients, apart from delivering the meals. The interviews also facilitated the exploration of whether participants’ experiences with clients and delivering the service might have changed during the first national UK lockdown, and their perceptions of the implications that a new wave of infections might have on them personally, their clients, and the MoWs service more generally.

All interviews were conducted by the second author, an experienced qualitative researcher. No prior relationship had been established between the interviewer and participants. Participants were asked to report their role, and how many years they had been working for the service. Notes were kept during the interview to help probe for more information and verify responses at transcription. At the end of the interviews, a summary of main points was provided to participants, which helped to confirm accuracy of responses (Korstjens & Moser, 2018). Interviews were transcribed verbatim and anonymised; transcripts were compared with recordings and field notes to verify credibility.

### 2.3 Data analysis

Data were analysed thematically (Braun & Clarke, 2006). The second author read through the 18 transcripts and coded them inductively, which involved initial coding of the data into broad codes. This was followed by a process of secondary coding, where data were analysed line‐by‐line to create specific codes. The fifth author independently coded 30% of the transcripts (*n* = 5) to ensure rigour of the coding process (Korstjens & Moser, 2018). Any discrepancies were discussed, after which the coding process was refined; the second author then coded all transcripts using this codebook. Any new codes were noted, discrepancies were discussed and the codebook was refined. The codes were organised into themes and sub‐themes, and further reviewed by the team to ensure coherence within and across themes (Elliott, 2018).

NVivo software (version 12.0, QSR, Southport, UK, 2018) was used to facilitate the coding. Themes and sub‐themes are illustrated with representative quotations from participants (indicated as P1‐P18 and driver (D)/office‐based role (O)). Additional quotations are provided in Table S2.

### 2.4 Ethical considerations

The study was approved by the School for Policy Studies Research Ethics Committee at the University of Bristol (SPSREC/19‐20/115) and complied with the European General Data Protection Regulation. Participation was voluntary. Participants were provided with an information sheet describing the aims and processes of the study, provided written informed consent prior to interviews and received a £10 gift voucher as a token of appreciation.

## 3 FINDINGS

Interviews were conducted with 18 MoWs employees (Table 1). Participants had worked with MoWs for an average of 10.7 years, and all were paid employees. Findings are presented under five themes (Table 2).

**TABLE 1:** Participant characteristics

	Local authority 1 (urban) (*n* = 13)	Local authority 2 (semi‐urban) (*n* = 5)
Sex		
Male	5 (38.5)	1 (20.0)
Female	8 (61.5)	4 (80.0)
Role in the service		
Driver	8 (61.5)	4 (80.0)
Customer advisor	2 (15.4)	—
Manager/service coordinator	1 (7.7)	1 (20.0)
Operations Officer	1 (7.7)	—
Rounds coordinator	1 (7.7)	—
Years of working in the service (mean and standard deviation)	9.1 (12.8)	14.9 (5.1)

Note

Numbers represent *n* (%), unless otherwise stated.

**TABLE 2:** Themes and sub‐themes resulting from the thematic analysis

Theme	Sub‐theme
Benefits to clients	‐ Encouraging clients to eat and keep physically active ‐ Carrying out chores for clients ‐ Carrying out welfare checks ‐ Identifying, addressing and reducing isolation and loneliness ‐ Promoting independence and rehabilitation following hospital discharge
Benefits to employees	‐ Sense of pride, giving something back, being caring ‐ Reciprocal positive relationships with clients
Being the fourth emergency service	‐ Being the first responders ‐ Getting food to clients during adverse circumstances
Wider benefits to the community	‐ Reducing pressures on families
Challenges faced by the service	‐ Organisational challenges ‐ Restrictions on time spent with clients
Challenges that emerged during the pandemic	‐ Demands on resources ‐ Impact on client and staff wellbeing ‐ Concerns about the future of MoWs, particularly in further lockdowns

### 3.1 Benefits to clients

#### 3.1.1 Encouraging clients to eat and keep physically active

Drivers reported that they encourage clients to eat as part of their role. However, this depended on the individual circumstances of each client, with some needing more encouragement than others. *You might go in to a gentleman who is in his 70s who is fit as a flea. So you are in there, meals down. They are fine. Go on to the next one who has dementia, it is a totally different ballgame. You plate the dinner. You take it to them, the cutlery. Explain, ‘there is the food, leave it for a minute, it is hot’* (P1/D). Most participants reported that promoting physical activity to clients is not part of their role, as they are *‘not trained in that’* (P5/O). However, some drivers discussed how they try to encourage clients to move more, which included walking to the table to eat, rather than bringing clients their meal on a tray, and inviting clients to take a wander around the house to *‘get them up and moving’* (P12/D).

#### 3.1.2 Carrying out chores for clients

Participants discussed how they carry out different chores for clients when they deliver meals, both inside and outside the home. Several indicated the importance of this, particularly for clients who had mobility difficulties. *Just general: check the gas meter, change the batteries in the clock, put the clock back to the right time. Find someone's glasses*. *Close windows, open windows… Just small things generally* (P15/D). Other chores included managing rubbish and recycling, changing lightbulbs and ensuring clients have easy access to the telephone.

#### 3.1.3 Carrying out welfare checks

Participants reported how, far more than delivering a meal, their role included carrying out welfare checks. It was argued this was a benefit stemming from MoWs being run by the LA, and not a private company. *I think the advantage of the City Council service over, let's say [private food delivery company], is that we offer that kind of safeguarding and interaction bit* (P11/D). As part of these checks, participants checked for safety concerns and household hazards, including trip hazards, whether the heating works, checking the fridge for old food and checking for gas leaks. In addition, participants from one LA had received training in medication prompting and therefore prompted clients to take their medication. However, participants in the other LA acknowledged this is a service they are unable to provide without receiving the appropriate training. Overall, welfare checks were considered *‘little things to us, but they are … vital to them’* (P3/D).

#### 3.1.4 Identifying, addressing and reducing isolation and loneliness

Social interaction is also an important part of the MoWs service and a way to identify, address and reduce isolation and loneliness in clients, as it helps with *‘taking the anxiety levels down a notch’* (P18/D). Social interaction was deemed particularly important during lockdown; participants reported that their clients were feeling extremely lonely as they were not being visited by family members *‘because of the fear of COVID and passing it on’* (P1/D). This participant also highlighted that for some clients, they are the only person they see on any given day. Y*ou go in there and you see their faces light up… they are absolutely delighted to see you… because you might be the only person that they see daily* (P1/D). As a result, and in response to each client's presenting needs, one MoWs manager described how they signposted clients to other services, which might offer support and opportunities to engage with others, for example online classes and groups being run by older people's charities.

#### 3.1.5 Promoting independence and rehabilitation following hospital discharge

Participants also discussed how MoWs are important for promoting independence and enabling clients to *‘stay that bit much more independent in their own home’* (P3/D). In particular, the welfare checks conducted by MoWs drivers were perceived to support older adults to remain in their own homes rather than be admitted to residential care. One participant highlighted how this saves on healthcare costs. *On one level, we are saving the council thousands, given the fact people are staying in their own home… If you are a private client, it is around £6.60 you pay for meals on wheels… Residential care, you are looking at £5,000 to £7,000 per week* (P13/O). The service was also deemed important in promoting rehabilitation following hospital discharge. Some drivers noted improvements in clients’ health and activity over time after receiving MoWs post‐discharge. *We see a lot of people who come out of hospital and can't cook for themselves. So, over the weeks, you'll see them growing in strength and growing in confidence, and then they'll go back to cooking their own food* (P4/D).

### 3.2 Benefits to employees

#### 3.2.1 Sense of pride, giving something back, being caring

Participants overwhelmingly reported that they enjoy working for MoWs and that it was the social and caring aspects of the job that they found appealing, as well as giving them a sense of pride. This was particularly evident during the pandemic. *Recently I experienced a round of applause from one neighbour of an elderly person I was delivering to and countless well dones from strangers which, although embarrassing, filled me with a sense of pride* (P10/D). Some participants also highlighted how working for MoWs and supporting the most vulnerable helped them to give something back to their community. Being caring was also an important trait that some participants iterated. *Because of the nature of the drivers, they're all very caring…They don't like to see [clients] struggle…it's not something you can train them on really* (P14/O).

#### 3.2.2 Reciprocal positive relationships with clients

Participants discussed the rewarding nature of developing long‐term relationships with their clients. They highlighted that the time they spend talking with clients, particularly the ones they have been delivering meals to for many years, helps build relationships that are mutually rewarding: *because you do it every day, five days a week, week in week out, it's amazing how much you become, not a part of their lives, but feel that you really connect with them* (P18/D).

### 3.3 Being the fourth emergency service

#### 3.3.1 Being the first responders

Participants discussed how drivers delivering the meals are often the first responder to an emergency. This included finding participants on the floor, witnessing a health incident (e.g. a stroke), realising that clients’ homes had been broken into, and discovering that clients had died. They reported that in situations requiring an immediate response, drivers would stay with the client until help arrived and they would notify the MoWs office, who would then contact family members and the social services, to communicate these emergencies to all relevant parties. Participants also reported on how they work jointly with other emergency agencies, including the fire service (e.g. to install smoke alarms), the police and medical emergency departments to ensure clients’ wellbeing. One participant highlighted that a benefit of MoWs being coordinated and delivered by the LAs is that they have direct access to social services. *I guess one of the benefits of us being in the council is it is very easy for us to coordinate with other agencies… We have got access to the social service system here with immediate points of contact, which I kind of get the impression maybe community groups would struggle with. So I do think it is something that should stay within the council* (P13/O).

#### 3.3.2 Getting food to clients during adverse circumstances

Participants reported how they are *‘a lifeline’* (P12/D) and go above and beyond to continue delivering meals to clients during adverse circumstances, such as the pandemic and periods of extreme weather. One participant described that MoWs was perceived by one client as the ‘fourth emergency service’. *I never forget a classic night when it was really bad snow and I delivered to one lady and she was just like… She was nearly in tears, she said, ‘I really thought I wasn't going to see anybody today or get any meals. You feel like the fourth emergency service’* (P12/D).

### 3.4 Wider benefits

#### 3.4.1 Reducing pressures on families

Both the deliveries, and the checks on clients, were perceived to relieve pressures on families and/or carers, who might not only be unable to provide meals, but also immediate support for their vulnerable relatives. This was particularly the case during the pandemic, which prevented families from caring for their relatives due to travel restrictions. *Especially during COVID. At least the families were reassured that somebody was going in to check on their relatives and that they were getting something to eat* (P3/D).

### 3.5 Challenges faced by the MoWs service

#### 3.5.1 Organisational challenges

Participants expressed concerns about the service being under ongoing threat of closure and highlighted that MoWs will become more essential in the future, as the ageing population increases. Funding was identified as an important issue for the service to continue, with some participants highlighting the need for the national government to acknowledge that MoWs provide more than just meals. *We simply don't have the infrastructure to meet the demand… particularly as… Councils have literally been stripped to the bone of funding… now with COVID‐19 we can expect probably two, three years down the line even more cuts. I do feel if we can get this on a national level, whether it could be given the respect and appreciation it probably deserves, the meals service* (P13/O). Reduced resources had led one LA to outsource their administration to external private companies. This raised concerns about drivers feeling less supported by the new administrative teams. Several participants highlighted the importance of understanding clients’ individual needs and the local context, and that this is sometimes difficult for outsourced administration teams to achieve. *The admin part of the team has been outsourced… you get a lot of complacency within the service. I think, as a team with [name of Council], we really knew our clients… for me, I don't always feel – and the drivers don't always feel –entirely supported* (P5/O). Participants also largely reported that the service does not receive sufficient publicity, as many people allegedly do not know that MoWs still exist, or that the service goes beyond meal delivery to support vulnerable individuals. Another perceived misconception of the public, which some participants acknowledged, is that MoWs are only intended for older adults. *‘Making sure our service was recognised again, and the value of it’* (P16/O) was described as one of the biggest challenges MoWs face.

#### 3.5.2 Restrictions on time spent with clients

Participants discussed how the time available to deliver meals to their clients is restricted, as meals need to be delivered within deadlines. This had implications for interactions with clients, and was often a cause of upset for drivers, who found it *‘hard to deliver a meal and not stay for longer to chat knowing that most of the day the clients will be lonely in the current lockdown’* (P10/D). Drivers seemed to overcome this restriction by alternating the amount of time they spend with different clients every day, doing administrative tasks beforehand and multi‐tasking while at a client's home, e.g. looking around the house for hazards while plating a meal. However, several suggested that more time should be spent with clients. *At least about 20 min, half an hour would probably be nice. Some people do need that little bit more time* (P3/D).

### 3.6 Challenges that emerged during the pandemic

#### 3.6.1 Demands on resources

Participants reported an unprecedented demand for the service during the first COVID‐19 lockdown. This was compensated to an extent by redeploying staff, hiring more vehicles and increasing orders with food suppliers. However, demand for drivers was still evident, particularly if existing drivers fell sick and needed to self‐isolate. Demand on personal protective equipment (PPE) also increased and PPE was not always available during the first lockdown. Nevertheless, the service seemed to go beyond expectations to produce contingency plans and carry on throughout the pandemic. *I mean, we've got our staff. Some of our staff would do overtime as well*. *I cover rounds in emergencies. So, yes, I’m sure we'd find people that would be able to do it* (P16/O).

#### 3.6.2 Impact on client and staff wellbeing

A direct repercussion of the increased demand for MoWs during lockdown was that the time available to interact with clients was further reduced, which compromised drivers’ ability to conduct welfare checks. This was particularly the case when drivers had to make doorstep deliveries. The use of PPE further compromised time spent with clients, as precious time was taken up with putting equipment on each visit. Participants also perceived that PPE made communication with clients challenging. *The masks, one woman was very upset that I was wearing a mask…I said, “Look, have you been watching the news?” “Yes,” and I said, “Look, I’m still here. I’m under the mask, but it's really here just to keep you safe and make sure we don't, sort of, pass on anything to you.”In time, they've gotten used to it* (P11/D). Participants also reported increased concerns about their own mental and physical wellbeing, particularly at the beginning of the pandemic, and how they were anxious about catching COVID‐19 and spreading it to their families, but also their clients. The situation was particularly stressful for those participants who did not have access to PPE at the beginning of the lockdown, and this was ongoing for those who continued to enter clients’ homes and did not conduct doorstep deliveries. Some participants also mentioned how seeing their clients during the lockdown impacted on their own mental wellbeing. *Definitely mood‐wise, I get a lot of people now in tears, saying they're lonely, they're frightened. That's hard* (P7/O).

#### 3.6.3 Concerns about the future of MoWs, particularly in further lockdowns

Some participants expressed concerns about how MoWs will cope in the case of continuous lockdowns. Specific concerns revolved around increased demand, food sourcing and human resources, particularly *‘if our drivers start having to self‐isolate themselves’* (P14/O). Nevertheless, they remained positive that the service would find a way to continue supporting the most vulnerable, as *‘I don't think there's anything we wouldn't try to do for these people’* (P12/D). Specific contingency plans included employing casual staff and making sure they had sufficient stocks of PPE. Nevertheless, some participants were well‐aware that the future effectiveness of their service depended highly on the support received from LAs and the national government. *I think the challenge, when life does return to normal, is how those things are perceived by the City Council themselves and by national government in terms of how they fund it and where they continue to support it through additional funding, acknowledging the fact that it's not simply a food delivery service* (P11/D).

## 4 DISCUSSION

This study exploring the experiences of MoWs employees in two English LAs highlights the wider benefits of the service, with participants drawing attention to the emergency response the service entails to ensure clients’ wellbeing. Participants provided important insights on the challenges they encounter, including COVID‐specific challenges, and those that the service faces more widely. These findings add to the limited global literature around the benefits and challenges of MoWs, and shape a crucial formative research evidence base for the development of interventions and policies aiming to support vulnerable individuals, particularly during continuing lockdowns, but also beyond the COVID‐19 pandemic.

Participants reported that MoWs bring a variety of benefits to clients that extend beyond nutrition. These include carrying out chores and welfare checks for clients. This is in agreement with a US study, which found that MoWs drivers perform essential tasks around clients’ homes, raise attention to potential hazards and conduct safety checks (Thomas et al., 2020). Other studies have highlighted the role of MoWs in reducing isolation and loneliness, as well as in promoting independence and decreasing the need for residential care and healthcare costs, which was confirmed by the current findings (Thomas et al., 2016; Thomas & Mor, 2013). Of note, participants in the current study mentioned that for some clients, MoWs drivers are the only people they see on any given day. The benefits of social interaction that MoWs clients receive have also been reported in earlier studies (Grant & Jewell, 2004; Timonen & O'Dwyer, 2010). As housebound individuals are likely to report depressive symptoms (Sirey et al., 2008), these findings suggest that MoWs are an important community resource to reduce social isolation of housebound vulnerable individuals.

A unique finding of the current study was that some drivers encouraged their clients to move more, even though promoting physical activity is not part of the service's remit. Considering that physical activity among housebound individuals is challenging (Szanton et al., 2016), MoWs could serve as an important community intervention to promote movement in this population, with careful planning to account for clients’ individual circumstances. In addition, only those participants who had received training in medication prompting were allowed to remind their clients to take their medications. Expanding this training for all LAs offering MoWs might provide a crucial lifeline for clients with cognitive decline, who are a considerable proportion of MoWs recipients (Altshuler & Schimmel, 2010; Xu et al., 2010). Further, we found that MoWs drivers are often the first responders in emergencies, which supports research among recipients of MoWs in Ireland (Timonen & O'Dwyer, 2010). Another unique finding of the current study is that the MoWs service collaborates with emergency and social services to ensure that vulnerable clients are cared for. The service also goes ‘above and beyond’ to continue delivering meals in adverse circumstances, such as during periods of extreme weather and the COVID‐19 pandemic, suggesting its crucial role in protecting the most vulnerable.

Our findings regarding the benefits of MoWs on employees themselves add to the limited evidence base and corroborate those of a US qualitative study (Thomas et al., 2020). We found that working in this service exerts a sense of pride for giving something back to the community. Participants also described the development of reciprocal and caring relationships with their clients, which are equally rewarding to themselves as to their clients. Even though all participants in the current study were paid employees, these findings are important for other MoWs settings globally that rely heavily on volunteers; the reported perceived benefits of working for the service should be highlighted to incentivise recruitment and raise awareness that this role involves more than just delivering a meal.

Several challenges faced by MoWs emerged from the current study. Participants highlighted that there are ongoing concerns about budget cuts and service closures, which would be particularly concerning if an increasingly ageing population led to significant service demand (Campbell et al., 2015). This is particularly significant, given that a variety of ageing and environmental factors, including physical challenges, difficulties around food consumption and access and use of transport are common in older adults, and identified as contributing to their need for food assistance (Lee et al., 2005a; Warren et al., 2020). Despite their projected benefits in reducing healthcare and long‐term care costs (Meals on Wheels America, 2020; Thomas & Mor, 2013), MoWs are suffering budget cuts globally, or do not receive sufficient funding to keep up with the need for the service; this hinders their ability to deliver community services to the most vulnerable and their capacity to respond to increased service demand (Gualtieri et al., 2018; Winterton et al., 2013). Our study adds to these findings by showing that this was particularly the case during the lockdown, when demand increased and having sufficient human resources was often a challenge. Even though outsourcing of administrative duties can form part of successful MoW delivery models and might relieve budget and human resource pressures that LAs face (Winterton et al., 2013), our findings draw attention to potential pitfalls of outsourced administrations. Notably, there was a concern about outsourced teams lacking awareness of the local context and the specific circumstances of clients, which might contribute to MoWs drivers feeling less supported to carry out their role. Also, keeping the administration and delivery of MoWs ‘in‐house’ seemed to facilitate a rapid and coordinated response in emergency situations, between all the services that might be needed to ensure a client's wellbeing. This finding should be studied further, but it indicates the importance of keeping the service local. The current study also highlighted that MoWs do not receive sufficient publicity in England, hindering awareness of the service among people who might need it the most. This supports findings from an earlier US study among providers of nutrition programmes for older adults, which identified that misconceptions and lack of awareness of such programmes are important challenges to outreach and meeting the needs of individuals who live in the community and are in need of care and support (Lee et al., 2005c). It is therefore important to raise awareness of the existence of MoWs and the service's wider preventative value via appropriate framing of publicity resources (Papadaki et al., 2021).

It is noteworthy that participants in the current study raised concerns about how time available with each client is restricted, and had reduced further during the pandemic. Having sufficient time to interact with clients beyond the meal delivery itself is an important element of the service's social nature and is crucial to carry out welfare checks (Winterton et al., 2013). Earlier research has demonstrated the challenges that time pressures place on the amount and quality of social interaction MoWs clients experience (Frongillo et al., 2010; Timonen & O'Dwyer, 2010). Despite the rewarding nature of the job, participants reported many challenging situations they encounter daily that can have a negative impact on their mental health. The need to support MoWs employees’ wellbeing is therefore crucial so that they continue providing this important service (Papadaki et al., 2021).

### 4.1 Implications for practice and policy

Despite the small‐scale, exploratory nature of this study, our findings could have important implications for practice and policy in England, but also globally, at least in settings where MoWs services are commissioned, organised and delivered in a similar manner to the LAs participating in the current project. The wide range of benefits exerted by MoWs should be acknowledged nationally, recognising MoWs as an emergency response service with a crucial preventative role in maintaining the wellbeing and independence of housebound older individuals. Findings also highlight the benefits gained from drivers building long‐term relationships with clients and getting to know their individual needs and health concerns. This has implications for how MoW services are staffed and resourced. The challenges faced by the service, particularly during the pandemic, indicate the need for national policies to protect MoWs through increased support and funding to LAs, which would allow the continuation of the service nationally. This continuing governmental support could help MoWs allocate appropriate human resources so that time spent by drivers with each client is not compromised.

### 4.2 Strengths and limitations

To our knowledge, this is the first study to explore the perceptions and experiences of MoWs providers in England, with regards to the service's benefits, challenges and experiences during the first national UK lockdown caused by the pandemic. Considering these issues in the light of service providers’ perspectives is an important step in understanding how MoWs should be targeted, and how this can translate to policies, to meet the needs of individuals who require food assistance (Lee et al., 2005b). As such, our findings contribute to the international evidence base reporting on MoWs, while providing novel insights about the importance of the service in supporting vulnerable individuals during the current pandemic. Some limitations should be noted. Due to the rapid nature of this research, we recruited employees from two LAs only, hindering the study's generalisability. Nevertheless, we recruited participants occupying different positions and the participating LAs serve urban and semi‐rural areas, which helped gain a wide range of insights. Future studies should ideally recruit participants across the four UK nations (England, Scotland, Wales and Northern Ireland) to obtain more representative views and experiences. In addition, the sample size was relatively small, and we cannot exclude the possibility of self‐selection bias. However, the study took place while COVID‐19 restrictions were in place, which might have made it challenging for employees with competing priorities to participate.

## 5 CONCLUSIONS

This small‐scale study highlights the wider benefits of MoWs and the ongoing challenges services face. It also highlights unique challenges introduced by the COVID‐19 pandemic, which might hinder the service's performance and beneficial outcomes in a combined situation of continuing lockdowns, increased number of individuals needing to self‐isolate, and insufficient financial support to meet the demand. Due to the aforementioned limitations, our findings are exploratory in nature; nevertheless, they suggest that health and social care national policies should prioritise the revival, re‐introduction and enhancement of MoWs, by supporting LAs to meet increased demand, particularly during the pandemic. These recommendations will help ensure that the most vulnerable individuals in the community continue to be supported, not only during the pandemic, but beyond.

## CONFLICTS OF INTEREST

The authors declare no conflicts of interest relevant to this study.

## AUTHOR CONTRIBUTIONS

AP conceived the study, with input from PW, KST, AC and MA; KST and EAG contributed to the study design; BA collected the data; BA, AP and PI analysed the data, with input from all co‐authors; AP led the drafting of the manuscript. All authors provided critical input, reviewed the manuscript for important content, take responsibility for the contents of this article and approved the final version submitted for publication.

## ETHICS INFORMATION

The study was approved by the University of Bristol, School for Policy Studies Research Ethics Committee (SPSREC/19‐20/115).

## Supporting information

Supplementary Material

## Data Availability

The interview guides used to collect data, as well as processed data, are available in the supplementary material of this article. Other data that support the findings of this study are available from the corresponding author upon reasonable request.
