# Supplementary material for: ‘It’s not just about the dinner; it’s about everything else that we do’: A qualitative study exploring how Meals on Wheels meet the needs of self‐isolating adults during COVID‐19
Source: Health Soc Care Community. 2021 Nov 12:10.1111/hsc.13634. Online ahead of print. doi: 10.1111/hsc.13634 (PMC8652984; doi:10.1111/hsc.13634)
Supplement: Supplementary file 1 — Supplementary Material [file HSC-9999-0-s001.docx]

**Table S1:** COREQ (Consolidated criteria for reporting qualitative studies) checklist

| **No** | **Item** | **Guide questions/description** | **Page** |
| --- | --- | --- | --- |
| **Domain 1: Research team and reflexivity** | | | |
| **Personal Characteristics** | | | |
| 1. | Interviewer/facilitator | Which author/s conducted the interview or focus group? | 6 |
| 2. | Credentials | What were the researcher's credentials? *E.g. PhD, MD* | 6 |
| 3. | Occupation | What was their occupation at the time of the study? | 6 |
| 4. | Gender | Was the researcher male or female? | 1 |
| 5. | Experience and training | What experience or training did the researcher have? | 6 |
| **Relationship with participants** | | | |
| 6. | Relationship established | Was a relationship established prior to study commencement? | 6 |
| 7. | Participant knowledge of the interviewer | What did the participants know about the researcher? e*.g. personal goals, reasons for doing the research* | 6 |
| 8. | Interviewer characteristics | What characteristics were reported about the interviewer/facilitator? e.g. *Bias, assumptions, reasons and interests in the research topic* | 6 |
| **Domain 2: study design** | | | |
| **Theoretical framework** | | | |
| 9. | Methodological orientation and Theory | What methodological orientation was stated to underpin the study? *e.g. grounded theory, discourse analysis, ethnography, phenomenology, content analysis* | 6-7 |
| **Participant selection** | | | |
| 10. | Sampling | How were participants selected? *e.g. purposive, convenience, consecutive, snowball* | 6 |
| 11. | Method of approach | How were participants approached? e*.g. face-to-face, telephone, mail, email* | 6 |
| 12. | Sample size | How many participants were in the study? | 6 |
| 13. | Non-participation | How many people refused to participate or dropped out? Reasons? | 6 |
| **Setting** | | | |
| 14. | Setting of data collection | Where was the data collected? e*.g. home, clinic, workplace* | 6 |
| 15. | Presence of non-participants | Was anyone else present besides the participants and researchers? | N/A |
| 16. | Description of sample | What are the important characteristics of the sample? *e.g. demographic data, date* | 6, 7, Table 1 |
| **Data collection** | | | |
| 17. | Interview guide | Were questions, prompts, guides provided by the authors? Was it pilot tested? | 6 |
| 18. | Repeat interviews | Were repeat interviews carried out? If yes, how many? | N/A |
| 19. | Audio/visual recording | Did the research use audio or visual recording to collect the data? | 6 |
| 20. | Field notes | Were field notes made during and/or after the interview or focus group? | 6 |
| 21. | Duration | What was the duration of the interviews or focus group? | 6 |
| 22. | Data saturation | Was data saturation discussed? | 6 |
| 23. | Transcripts returned | Were transcripts returned to participants for comment and/or correction? | 6-7 |
| **Domain 3: analysis and findings** | | | |
| **Data analysis** | | | |
| 24. | Number of data coders | How many data coders coded the data? | 7 |
| 25. | Description of the coding tree | Did authors provide a description of the coding tree? | 7 |
| 26. | Derivation of themes | Were themes identified in advance or derived from the data? | 7 |
| 27. | Software | What software, if applicable, was used to manage the data? | 7 |
| 28. | Participant checking | Did participants provide feedback on the findings? | 6-7 |
| **Reporting** | | | |
| 29. | Quotations presented | Were participant quotations presented to illustrate the themes/ findings? Was each quotation identified? e*.g. participant number* | 7, Results section |
| 30. | Data and findings consistent | Was there consistency between the data presented and the findings? | Results section |
| 31. | Clarity of major themes | Were major themes clearly presented in the findings? | Results section |
| 32. | Clarity of minor themes | Is there a description of diverse cases or discussion of minor themes? | Results section |

**Appendix S1:** Interview guide for drivers

1. Could you tell me about your professional background?

[**Prompt for**: qualifications; settings worked in; types of service users worked with; length of time working with older people?)

1. How did you come to be working as a driver for MoW? [**Prompt for**: length of time working in MoW; exact role (paid or volunteer); what motivated you?]
2. How do you find the experience of working with clients in MoW? [**Prompt for**: enjoyment; challenges and benefits; what do you value about the service?] [**Follow-up:** what things would you change about your role and the service provided?]

IF VOLUNTEER:

- What do you enjoy about volunteering? What’s the best thing about being a volunteer for this service?

1. Please describe the orientation or training that you received when you became a driver for this programme.

- What were the most important aspects of this training, in your opinion?
- What were you trained to do if you were unable to deliver the meal (i.e., a client does not come to the door)?
- Did your training include anything about what to do if you notice that something is wrong or different with the client?

1. Could you please describe your route? [**Prompt for**: how many clients you deliver meals to; areas delivering meals to; how long it usually takes; how often you deliver meals; same routes on a regular basis?]

Now think of the time before COVID-19 (I will ask you about that later)…

1. Can you tell me about the people you deliver meals to? [**Prompt for**: age, living arrangements, functional limitations, why you think they need Meals on Wheels?]
2. From your experience, or from what clients tell you, what are the benefits of the service to the people who receive the meals? [**Prompt for**: what do clients value about MoW?]
3. How do you think your work promotes clients’ wellbeing? Can you provide examples?
4. Can you please describe a typical meal delivery interaction? [**Prompt for**: how much time you typically spend with clients, do some clients receive more of your time due to their need/request, do you provide any assistance to them in addition to handing them a meal, like opening containers, placing in refrigerator, setting up the meal?) [**REMIND respondent** not to divulge personal information or information that could potentially identify the older person]
5. Can you please tell us whether you provide any other assistance to your clients, beyond the meal delivery? [**Prompt for:** welfare checks, remind them of medication, chores around the house, call someone for them?] [**Follow-up:** did you receive any specific training to prepare you for this wider role?]
6. Can you tell us whether you talk with your clients at all about physical activity, and in which context that would be? [**Prompt for:** walking around the house; time spent sitting; gardening?] [**Follow-up:** did you receive any specific training to prepare you for this wider role?]
7. How do you feel your interactions and assistance you provide to clients might differ if working with different types of needs? [**Prompt for**: dementia; complex physical and mental health needs, people at the end of life, carers of older people].
8. What would you say are the challenges in your job and the MoW service more generally? [**Prompt for:** lack of governmental funding - sustainability; the number of people in need of the service – demands on the service; available resources; challenging clients; challenging incidents?]
9. Do you look for safety concerns in the clients’ homes? If you notice a safety concern, what do you do?

- What kind of safety concerns do you normally check for?
- Is there a standard safety assessment or guidelines you use (i.e. to look for objects on the floor, check surroundings, etc.)?
- [**Follow-up:** did you receive any specific training to prepare you for this wider role?]

1. Can you provide an example of a time when you noticed something wrong or different about a client?

- What did you do?
- What happened next?

IF NOT ADDRESSED:

- Is there a formal process for telling the staff when you observe a safety concern? If you observe a safety issue, is there a follow-up process? Do you know what happens next?

1. Now please think of the recent lockdown period. In what way might your experience with delivering the service change? Can you provide some examples? [**Prompt for:** for the service; change in interactions with clients; interaction according to different needs of clients?]
2. What do you think the implications of a new wave of COVID-19 infections (and potential lockdown) be on the MoW service, your experience as a person delivering meals, and the needs of your clients? [**Follow-up:** do you have any suggestions on how these could be overcome?]
3. Is there anything else you would like to say about your interactions with MoW clients or the benefits of the service that hasn’t been covered?

**Appendix S2:** Interview guide for Meals on Wheels employees with office-based roles

1. Could you tell me about your professional background? [**Prompt for**: qualifications; settings worked in; types of service users worked with; length of time working with older people?)
2. How did you come to be working as a customer service adviser for MoW? [**Prompt for**: length of time working in MoW; what motivated you?]
3. How do you find the experience of working with clients in MoW? [**Prompt for**: enjoyment; challenges and benefits; what do you value about the service?] [**Follow-up:** what things would you change about your role and the service provided?]
4. Please describe the orientation or training that you received when you became a customer service adviser for this programme.

- What were you trained to do?
- What were the most important aspects of this training, in your opinion?
- Did your training include anything about what to do if you notice that something is wrong or different with the client?

Now think of the time before COVID-19 (I will ask you about that later)…

1. Can you tell me about the people your programme serves? [**Prompt for**: how many clients; in what geographical areas; age; living arrangements; functional limitations;social support; why you think they need Meals on Wheels?]
2. Please describe how individuals typically are referred, screened, and assessed for meal services? [**Follow-up:** From whom do you receive the greatest number of referrals (healthcare, self-referred, family, community organisations)?]
3. How long does it typically take to start meal service from the time that a client is referred or service is requested?

- Do you have a waiting list? How is the waiting list managed (clients prioritised for services)?
- How do you feel about the way the waiting list is managed? Is there a different way you would prefer to manage it?
- What is the size of your waiting list on average?
- How long are participants typically on the waiting list?
- What impact did the COVID-19 outbreak have on this process [**Prompt for:** number of clients, assessment, waiting list]

1. Do you conduct client reassessment? When and why would you go out to reassess clients?
2. Please describe your delivery system.

- How many volunteer and paid staff do you have in your programme?
- Please describe the typical meal provided to clients (hot, frozen, chilled)?
- What about your delivery schedule/frequency (daily, weekly, weekend)?
- What is your main source of funding for meals (fee for service, other?)?

1. From your experience, or from what clients tell you, what are the benefits of the service to the people who receive the meals? [**Prompt for**: what do clients value about MoW?]
2. How do you think your work promotes clients’ wellbeing? Can you provide examples?
3. We understand that some MOWs offer additional services beyond meals.Can you please tell us whether you provide any other assistance to your clients, beyond the meal delivery? [**Prompt for:** welfare checks, remind them of medication, chores around the house, call someone for them; referral to a partnering organisation or social service?] [**Follow-up:** When and how are referrals for additional services made?]
4. Can you tell us whether you talk with your clients at all about physical activity, and in which context that would be? [**Prompt for:** walking around the house; time spent sitting; gardening; referral to community programmes about physical activity or exercise?]
5. Are there additional supportive services your organisation would like to provide (assuming availability of funds)?

- What would be needed in order to provide those services?

1. How do you feel your interactions and assistance you provide to clients might differ if working with different types of needs? [**Prompt for**: dementia; complex physical and mental health needs, people at the end of life, carers of older people].
2. What would you say are the challenges in your job and the MoW service more generally? [**Prompt for:** of governmental funding - sustainability; the number of people in need of the service – demands on the service; available resources; challenging clients; challenging incidents?]
3. Do you look for safety concerns in the clients’ homes? If there is a safety concern, how is it managed?

- What kind of safety concerns do you normally check for?
- Is there a standard safety assessment or guidelines you use (i.e. to look for objects on the floor, check surroundings, etc.)?

1. What are volunteers expected to do if they observe new or different problems, unusual behaviors, or changes in their clients’ situation (such as environment or social supports)?
2. Can you provide an example of a time when you noticed something wrong or different about a client in your interactions with them (or when a driver delivering the meal did so and reported it to you)?

- What did you do?
- What happened next?

IF NOT ADDRESSED:

- Is there a formal process you follow when drivers report a safety concern to you?
- What is done with this information?
- What do you see as the strengths of this system?
- Whatabout any challenges?
- What opportunities do you see for improvement?

1. Does your organisation have any relationships with healthcare providers or insurers?

- If so, what types of relationships do you have?
- Tell me about any healthcare providers in your area that you think would be interested in partnering with MoW to address the health and social support needs of the clients.

1. Now please think of the recent lockdown period. In what way might your experience with engaging with clients change? Can you provide some examples? [**Prompt for:** demand for the service; change in interactions with clients; interaction according to different needs of clients?]
2. What do you think the implications of a new wave of COVID-19 infections (and potential lockdown) be on the MoW service, your experience as a person engaging with clients and their carers, and the needs of your clients? [**Follow-up:** do you have any suggestions on how these could be overcome?]
3. Is there anything else you would like to say about your interactions with MoW clients or the benefits of the service that hasn’t been covered?

Table S2: Additional quotations from the themes and sub-themes resulting from the thematic analysis

| **Theme** | **Sub-theme and quotations** |
| --- | --- |
| **Benefits to clients** | **- Encouraging clients to eat and keep physically active**  *Before you know it, they’ve forgotten that their meal is on the table. So that would be on the notes, “Encourage to eat.” That might come from your checks or that might, again, come from the family. Again, if somebody is not feeling 100%, it’s just saying, “Well why don’t you just have a little bit?” I always say to them, “Why don’t you just have a bit of the pudding?” “Oh, that’s the best bit anyway.” You know, just something so they’ve eaten something* (P12/D)  *Other clients you know that are unable to communicate, we go to one service user, he is deaf and he is blind, so we go in, we plate up his meal, get his cutlery out, put the cutlery in front of him, the food in front of him, and then also he likes to have a cup of tea and a glass of water with his meal as well. So the driver would go and prepare that for him* (P13/O)  *We say to the drivers, at least remove the lid because the smell of the food will probably encourage them, perhaps, to eat their food* (P16/O)  *If you're not feeling that hungry, which lots of them aren't, they might not bother. But if you actually put the meal in front of them, hopefully, they are more inclined to eat it than if they have had to prepare it themselves* (P18/D)  *That is another thing I do. When I go in there I make sure they have eaten their dinner from the day before. Because if they are not they will put the hot dinner in the fridge. So I always check the fridges, make sure they haven’t put it in there from the day before. So you have to throw that food away and make sure that they are eating it* (P1/D)  *I mean, with people with dementia, you do have to prompt them to eat because you will give them a meal and they won't even realise that they're hungry. So, you do have to prompt them to eat and drink* (P3/D)  *We will go and get the knife or fork. We'll go and get the plate. We’ll plate it, actually bring it to them. That's encouraging them to eat* (P5/O)  *I might have to dish meals up, make sure they’re sat down, make sure I dish the meal up for them, take it to them, give them a knife and fork and make sure they start eating it, so it doesn’t get left* (P6/D)  *A lot of elderly people with dementia don’t even remember to eat, or have forgotten that they’ve eaten* (P7/O)  *A lot of the time it is because if they haven't got a meal put in front of them, they might not bother. So, it's lack of any will really, to prepare something* (P18/D)  *I’ve got to engage with her, to get her to eat because she doesn’t always try, you know, you’ve got to try and engage her to eat* (P10/D)  *We don't go in and cut up to everybody. Some people… it’s a doorstep delivery, especially during coronavirus. If they've expressed that they only want to be receiving the meal at the door, then that's what we do* (P5/O)  *Some customers just won’t eat it out of the foil so then we take the lids off, get them cutlery. Sometimes they want it plated up so we plate it up for them. Yes, we do that quite a lot actually* (P17/D)  *If they have got dementia… some of them will just forget to eat* (P18/D)  *In a sense, yes, I probably do. There was a lady who predominantly is always sat down… Always sat down. For quite a while I would, unless it says, “Must use the key safe”, I would ring the bell because I think it gets them up… Yes, in that sense, I would try to get them up and moving. I wouldn’t say I get them doing any exercises or anything like that. That’s not part of our role* (P12/D)  *No, not necessarily. To the extent that they would have that type of conversation, I mean where possible the drivers always try and ring the doorbell first, I know it is something small, just to make sure that the client inside gets up and moves out of the chair and that, but in terms of promoting physical activity themselves, no, not necessarily* (P13/O)  *Yes, definitely. If they have said that they have had difficulty moving around, or if they have come out of hospital, then we are always encouraging… normally with a walking aid. But saying, "Get as much exercise as you can. Go up and down the hall a couple of times," but move from the chair because we see it all the time where, if people don't keep mobile, then they just seize up completely. Especially because, if they have had a few falls, they get very anxious about falling again* (P18/D)  *Or if they have come out of hospital, then we are always encouraging… normally with a walking aid. But saying, "Get as much exercise as you can"* (P18/D)  *I’ve got one lady, we’re always talking about the weather. She always asks me whether it’s worth going out. I’m going, “Yes, you can go out, it’s nice.” She lives on the seafront… I say, “Go and have a nice walk, it’s lovely out there.”* (P2/D)  *I mean, sometimes you do say, "Have you been out today?" or, "Would you like to go for a walk?" Some actually do manage to get out because they have got carers, but not everybody can* (P3/D)  *Yes. And it’s not a bad thing in some cases because it does promote moving around, people getting out of their chairs, moving into the kitchen and that sort of thing* (P4/D)  *If they’re just sat there doing nothing then you do try to encourage them, even silly things like going from the living room to the kitchen, ‘why don’t you sit at the table and eat your meal rather than have it on a tray because then you’re moving’. It’s not much but it’s better than nothing* (P17/D)  **- Carrying out chores for clients**  *There are limits but predominantly, if there’s something they want helping with- Like they might say, “Could you go and get me this?” You would do it for them. I think they see that as such a nice thing* (P12/D)  *I’ve found I’ve done bits of shopping and stuff. I think with the meals on wheels service, I don’t think that will change* (P2/D)  *We have done, in the past, some shopping for some people. Some people do not just have their meals. They might have a loaf of bread and a pint of milk delivered. That’s quite common on the rounds now. Before, there has been a bit more of a wider shop for them* (P12/D)  *Some drivers, one would go off and pick up prescriptions for one of their service users because they have got no one. So we would be like, "Okay." It is not expected, but they would go and collect the prescriptions after work or drop off the post for others. Or even go and pick up some milk or some bread if the shop is just close by* (P13/O)  *We have got other drivers who would go in. Simple things like turning the television on, turning the volume down, turning the television off. Even the amount of times they get, "Can you turn this light switch on and off for me?", "Can you turn this light switch?" Simple things, day-to-day, mundane things, but just some of our service users really just can't do it. Either they are bed-bound or there is very little movement that they can do, which is why our drivers would go and support and sort them out* (P13/O)  *Sometimes if they have time they’d make a cup of tea. They’ve changed batteries in remote controls. They’ve re-tuned tellies. They’ve sorted out heating. Sometimes they’ve done the wrong switch so the driver will go in and check and if they can switch it on, that’s fine. If not, again, it’s reported to family* (P14/O)  *Somebody has a habit of taking all the phones off the hook, so they go around checking that the phones are on the hook, so the family can get hold of them* (P14/O)  *I think sometimes we're not supposed to do those things. With the clock, the lady asked me to change the clock time, and it was an old clock so the mechanism broke. And then I think, you know, they didn't like that. So I think technically we're not supposed to do those, but it's really difficult not to if somebody just wants you to reach something because they can't. And I make cups of tea as well* (P15/D)  *It’s going that little bit above and beyond, but knowing that you’ve still got other customers to go to. We can’t go in and make a cup of tea for every customer, but when it’s the odd one, then yes, we can do that* (P16/O)  *Sometimes, they’ve had problems with their telly and drivers have gone in and sorted out a problem with their telly. They’ve not really understood how to use the remote control. You know, so it takes that loneliness away because they’ve got their telly working for them* (P16/O)  *We’re asked to do them. If it’s a very simple task then yes, personally I’m quite happy to do it. If it’s shopping or housework it’s, “I’m really sorry. We haven’t got time to do that,” but if it’s something really simple. When I first started we used to have a lady and she used to get me to open a jar of pickles and open a bottle of wine for her. Things like that I’m quite happy to do for them if I’ve got the time to do it. I know one driver, they were getting him to put up curtain rails. He was doing it. It was like, “You really haven’t got time to do that.” But it’s one of those things* (P17/D)  *They often say, "Can you do me a favour?" and I say, "As long as you don't want me to vacuum the house, probably, yes." So, it's normally undo a bottle of bleach or something, or unscrew a jar or something. Those little things. Or get the bins in. Those sorts of things* (P18/D)  *To be honest, it’s all sorts you do. I post letters, rubbish goes out. You fix Sky, because you know Sky freezes on them. I can remember sitting there with one lady for about 10 or 15 minutes, I switched it off the plug and we had to wait for it to reboot. It’s all sorts you do. Changing clocks* (P2/D)  *Any rubbish building up. I’ve got one gentleman and every so often I have to say to him, “Your rubbish needs to go out because it’s starting to smell.” You do notice things building up. I’ve gone into some people’s places and they’ve got a saucepan on the cooker just burning away* (P2/D)  *Things like reading a letter or opening jars. That’s quite a common one, actually. Picking up post as we go in, because obviously quite a lot of them are unable to bend down and pick their post up. Changing batteries in clocks, I’ve done that. (Laughter) You know, just little things that they might be having trouble with. Oh, washing machines. Putting washing machines on. That’s another quite common one. I might help her out of bed and get her into a chair and ready to eat* (P4/D)  *It’s a lot of, like, “How are you doing?” and everything, you know, “How’s your day going? Are you okay? Can I help you with anything else? Is there anything else I can…?” you can pick the mail up off the floor if they’ve got a paper. You pick that up off the floor and just let them have it. So, it’s quite a lot to do. It’s not as easy as what everyone thinks it is* (P6/D)  *A couple of times, some of my clients have asked me. I check the fridge to make sure the stuff in there has not been in there and it’s in date. Two of my clients are likely to do that. It’s so quick, and then I say, “Oh, your milk’s gone out of date,” or something, and they say, “Oh.” I say, “Alright, look, when I finish work, because I’m coming past this way, I’ll go get you some and I’ll put it in the fridge for you.” So, I don’t have to do it, but it only takes five minutes* (P6/D)  *Some of it, I was able to just deal with myself. Like, if it’s the carpet turned up, I’ll just flatten that, you know what I mean?* (P8/D)  *Post is a regular thing, and, what else have I done? Yes, changed batteries in remote controls and all sorts, you know? It’s just, “Oh, do you think you could do this a minute?” Yes, just do it, definitely* (P9/O)  *Again, I think the drivers are so, so friendly. And they will do literally anything for the customer* (P7/O)  *It is just experience. And it is just natural. If you met anybody and they needed something done, and you have the time and the capacity to do it, I think it is just a natural thing that you will do it. If you haven’t got that feeling in a caring job I think you are in the wrong job, if I am honest* (P1/D)  *I’ve retuned radios, sorted out phone issues, post birthday cards and things. You know, we cover things they’ve dropped because, obviously, mobility is an issue for a lot of people* (P11/D)  *This will be a daily thing. I go in there. I put her meal on a tray. I put her rubbish and recycling out. I fill up her kettle. Normally, I have to put her cardigan for her, because she can’t do it herself… some days I have gone to find her phone for her, because she will put her phone down somewhere and she can’t find it. Anything that they can’t actually do. Change lightbulbs. Tune televisions in, when they need to be tuned every so often* (P1/D)  **- Carrying out welfare checks**  *You get into the house, you can see the conditions around, you can see whether they… you know, if they’re living quite well or if they’re able to cope. You can tell by the conditions. The majority of people are quite good, you know, and they’re quite clean. But there is a gentleman, he’s wandering around saying, “I feel sick, I feel ill.” So, I’m able to report that back to my bosses and then they can contact – he, this particular one, doesn’t have – I don’t think he has got family locally. It may be that they’re already aware of it but if you assume that they’re aware of it and they’re not, then you’re guilty of, you know, not doing your job basically* (P10/D)  *Yes, we do. As one example, if we deliver to a client where we have access to a key safe and don’t get an answer, we’re obliged to search the house to make sure that they haven’t come to any harm and are, sort of, in it, but not able to answer. So, occasionally, I have gone right through somebody’s house to make sure that they’re there and they’re actually out, and equally, whilst we’re delivering and we perceive there to be something that isn’t safe, we report that back to the office who then contact family or whatever the channels are to get somebody in to make sure that that’s dealt with* (P11/D)  *When we go in, with our wellbeing check, we make sure they are okay. If they say, “I don’t feel very well today.” You know, try to get a bit more background information out of them. If I’m really concerned, I will phone the office as soon as I get back in my van* (P12/D)  *We’ve been told to write anything which is significant on the sheets. I sometimes find if you’ve delivered to somebody at 12:00 o’clock. You might not get back to the office at 2:00 o’clock. A lot can happen in two hours. I think it proved that with the lady who fell over like she’d been broken into. That two hours could be crucial, so if I’m really concerned… I’m not saying everybody does this, because I can’t answer for everybody, but I do ring in. I say to the office, “I think, maybe, you need to ring the next of kin and say, “She’s not feeling very well* (P12/D)  *It is the right thing to do, and I think now more than ever people do need not just that 5-minute well-being check-in. It needs to be more developed; it needs to be a good 10, 15 minutes. Whether that needs to be with all customers, probably not. I think as we go through this process, we are just starting it up, so I think we will come to identify those who need the conversation maybe more than others. But for now we have just blanketed it as all customers are going to get this phone call* (P13/O)  *So the process for drivers is that they would contact the service desk here. So the customer service advisers, they would go through them, and the advisers here, they have a list of checks that they need to do. So whether that is, "Okay, first of all, can you check with neighbours? Whilst you are there, can you look through the letterbox? If you have a key safe, you need to go round the entire building, check the building." I think for me it is trying to- With the guys here, to understand that it is a professional service, and we need to make sure that we are doing all we can, which I know they do, to make sure that if anybody doesn't pick up, that we have done all we can to make sure that they are okay* (P13/O)  *We never used to do the welfare checks, like we do. I mean there was always the caring side, and they would always go above and beyond, but it wasn’t part of their job. Whereas now we do put more emphasis on checking on them, making sure that they’re okay. Looking out for signs of problems and things* (P14/O)  *Obviously if you see a socket hanging off the wall or something like that then you’d have to report it. Anything that doesn’t look right you report anyway just, as they say, to cover your back because if a relative went in and said, “This was wrong,” if I’ve already gone in and said to the office, “This is wrong,” then they can contact the relative. Again, it keeps them safe* (P17/D)  *I mean, at the moment, the clients know that you are just going to be in and out really, but whilst you are doing that, you're talking, you're checking, you're doing a few things. You would never actually sit down in a client's house, because your job is a moving job and they know that you're just going to be in and out. Which is good really, because you don't come completely enmeshed in their lives, but enough that you can spot differences and still feel that you make a difference* (P18/D)  *Sometimes you go in and somebody is very confused and really not themselves. Often, it's something simple, like a water infection. So, that would be definitely a case of not phoning in at the end of the day. That would be a question of phoning then and there to the office to advise them that Mrs so and so is definitely not herself and is very confused and disorientated and just not with it at all. So, you would phone the office and they would either, dependent on what next of kin arrangements there were, either phone next of kin or get in touch with the GP* (P18/D)  *Well, you keep on really, until something gets done. I mean, sometimes it's not quick because you have to take it into account, the client themselves might not want anything done. Might not be prepared to change anything. Even if you think, in your humble opinion, that they're not managing at home. If they don't agree with that or the family don't agree with that, then there's a limit to the amount, perhaps, that we can do. On the whole, if we have noticed something and it can be altered, then we pursue it until we can* (P18/D)  *Actually talk to them, engage with them. You can’t do a welfare check unless you actually engage with somebody and find out what is going on… You can tell. If you go and see somebody each day, you can tell just little subtle changes in their personality or the way they are, and you know that there is something wrong. Yes, you have to engage with them. Not just going in there, plating up a meal, and then getting out again.* (P1/D)  *Yes. We tell business support, and I would ask them to pass it on to next of kin, because they have got all the details. If family members are at the house when I am there I will have a word with them and I will voice my concerns. I always tell the drivers, if I am training them up, “If your gut feeling says there is something wrong, listen to that gut feeling. If you are wrong, you are wrong. It doesn’t matter. But you have still got to flag it up.”* (P1/D)  *Those are your main ones. Just to make sure that we do the welfare check. I can’t emphasise how important that is. If drivers are not prepared to go in someone’s house, even though it has obviously got to be safe, if they are not prepared to go in there then don’t come and do the job. We need people that are going to go in there and try and make abnormal normal. I think that is the whole crux of the matter. We have got to keep doing what we are doing. How can I put it? Don’t find excuses not to do the job so you can finish early. You have still got to get out there. You have still got to go in that person’s house. You have still got to see everything they are doing. That is why I say doorstep doesn’t work* (P1/D)  *What I tend to do, if I report anything back I say it by phone when we ring in at the end of our round and then I back it up with an email as well to make sure it has gone through. I always cc my manager in* (P2/D)  *We’ve had people come out, we’ve had councillors come out with us on a round and police community support officers come out with us as well. They’re all surprised at the end of the shift what we actually do and how well we know someone. Someone can say to me they don’t feel very well one day and I’ll always check up or ring through to the hospital and I check on how they’ve got on. Those sorts of things. They’re really surprised how well we know that person that we’re delivering too* (P2/D)  *It’s the daily contact with people. They have got somebody who they know is going to come in and check if they're okay. You go in and, as I say, you do know your clients. You know when something is not right because you see them every day* (P3/D)  *I think if you notice something that isn’t right, and you’re not happy about it, then we would report back. I’m trying to think of a good example. I mean, I’ve been into many, many houses that are really dirty and really unsanitary, and I might be the first one that goes in there, and then I would certainly report all of that back. Or, if somebody’s starting to look less well dressed or something like that, if it’s something out of the ordinary, I would report back* (P4/D)  *We've come across situations whereby, not so long ago, I went out on a round and the carer said, “I'll take the meal,” and we've made a point of saying, “No, actually. We're from the Council. Part of our duty is to actually see the client so that we can interact with them,” because we don't want to not see them. We like to go in and check them, but yes, so that's the sort of thing that we come across these days* (P5/O)  *Yes, so before COVID, it's difficult for me to… I would say that before COVID we were still going in to people that had already had care support. We were delivering. We were going in as that extra welfare check* (P5/O*)*  *Yes, drivers, the way that I train them is I say to them that “If you go into someone and you treat it like it's your grandparents or your parents, if you go in and that person is upset, or if there's anything that you think you're going to walk away from there and you're going to think, “God, that didn't seem right,” we don't want you to go home and worry about it. It's really important that you communicate that on so that someone else can deal with it. Maybe we get in contact with the next of kin, whatever the office choose to do, but whatever thing that bothers you, even if it's the slightest thing, just ring it through to the office, because we need to decide. We need to make the decision as to what we do with that information that you’re reporting.”* (P5/O)  *A man unfortunately had really deteriorated, he was completely incontinent, faeces and urine, and his house was just covered. It was awful. And he had no-one. He had no social worker, he had no next of kin. So, back at the office, they were able to call different companies, explain the situation, speak to the council, and say, “Look, the way this man is living is appalling, and someone needs to help.” And by doing that, he got the help he needed. He got allocated a social worker, that came out and sorted out his flat. And they got him to hospital and sorted out his medical issues that were causing it. But, if that was anyone else going in there, that wouldn’t even have been seen, it wouldn’t have been addressed. So, a lot of the times, we can really try and step in and change a person’s life, essentially* (P7/O)  *And a man who lost his wife to coronavirus, I do speak to him quite a lot. I do call him, I must admit, just to check in on him.* (P7/O)  *I rang the ambulance, and I was there for over an hour and a half, but the meal service, I go in some places and we’re the only people which go in there. I mean, all the council services are essential, but that is very essential because some of them don’t have carers, and they’re the only ones we’ll see, and if they don’t get their meals, they don’t get that* (P8/D)  *What most people get out of it is the welfare, the wellbeing check, the families, that’s what the families get out of it is the wellbeing check and they know that when they’re at work there’s somebody calling in on their elderly relatives and they’re being looked after. They do know that if there’s any problems during that day, they’ll get a phone call from us* (P9/O)  *Depending what it was, if it was a trip hazard, I’d sort it myself. If it’s something, I’d just move it off to one side and probably just make a little note on my sheets* (P6/D)  *Just making sure, as you walk through, that things aren’t in too much of a state for them. Some of them do, obviously, hoard things. You know, it’s just making sure they’re okay and they’ve got what they need in front of them. If there was a safeguard issue then, again, we would bring that back to the office. It’s not necessarily our job but- If it was something, just, minor then obviously you would help them move it out of the way or whatever. If it was something more, then you would let the office know and then they could contact the family or relevant services* (P12/D)  *But yes, wires, if holding is getting worse, you know and any broken sockets. Frayed carpets that could be a danger to the client or to them going in obviously. So yes, they’re just looking for everything* (P14/O)  *Rugs and furniture in the way. Previously, I have probably suggested it's a bit of a trip hazard. I mean, if there were wires across the floor* (P18/D)  *The first time you go in there you do a proper risk assessment. You do a sheet and then you give that into the office. But you still do it every day when you go in. Even if it is just… Well, it is just a quick look round. Trip hazards, if the rug has come up where they wander around. If they are a smoker, are they putting…? My father-in-law has got a habit of dropping his cigarettes on the floor. So if you know someone is a smoker you have a little check, make sure that they are not doing it. Just common sense. Anything where they could hurt themselves* (P1/D)  *We check for obvious things like trip hazards. Quite often, particularly if Social Services haven’t been involved, sometimes we can start with a client and they are hoarders. We’ve got a few hoarders. There are piles and piles of stuff everywhere in the house* (P4/D)  *So something we are introducing now, is the customer service advisers are doing weekly calls. So I have got 4 customer service advisers, and they are making 10 calls each a week to our service users, which first of all is just to speak to them, to get feedback of how they are finding the service at the moment, and then secondly just to have a conversation, to make sure they are all right, even if it is for 15 minutes of the day. Our customer service advisers, they have the time to do it, and I think, if we are going to go into a second lockdown and there are going to be more anxieties, I think this could be absolutely critical* (P13/O)  *The first time I was told this it shocked me. They say if they pass away and they don’t see anybody else, they want to be found* (P1/D)  *I think it’s essential… the drivers have come across people that have been attacked. They’ve come across them dead. So all these things, checking on the welfare, I think it’s essential these days. Especially when so many of them don’t seem to have family or people who bother with them, you know, it’s just sad* (P14/O)  *I quite often clear out old food from kitchens when I’m there, just for the safety issue. If they’ve half-eaten a meal from the day before, we clear it away and make sure that it’s gotten rid of* (P11/D)  *I’ve gone into places where you open the front door and you can smell gas. So, you go straight in, turn the gas stove off, or something like that* (P9/O)  *I have seen it so many times, when someone with dementia has got their plastic kettle and put it on the stove. Of course it has melted and caught flames. So you look for things like that* (P1/D)  *It’s things like that. I mean they’re not allowed to prompt the medicines or tablets or anything, at the moment. It is something that might change, yes. But I mean they would need training to do that and it would only be sort of prompting. Just saying, “Oh, can you remember to take your tablet” and stuff like that* (P14/O)  *We verbally prompt. Some of them you do have to take out the dosette box. Mainly, it’s verbally prompt. You have to be so careful because a lot of the families… You know the dosette boxes you can buy off eBay, a lot of the families put all the tablets in those, and we’re not allowed to do those. We’re not allowed to touch them because you don’t know what’s in there. We’re only allowed to deal with the ones that come from the chemist with the listed medication on* (P2/D)  *We have MedPrompt training because… sometimes we will prompt a client to take their medication at lunchtime* (P4/D)  *Prompting medication as well we do. We’re allowed to decant the medication from their dosette box, so they take those* (P2/D)  *If we get a customer saying, ‘Can you do me my medication?’, we’re not allowed to do that* (P7/O)  **- Identifying, addressing and reducing isolation and loneliness**  *So, I’ve got all these old people who aren’t speaking to anybody. The meal service is very essential and it’s a lot more than what meets your eye. It’s not just delivering meals. There’s people out there which are lonely. Some of them are okay, but if they didn’t get their meal, they wouldn’t see anyone* (P8/D)  *They’re lonely. There’s lots of lonely people in Bristol, there really is. Until I started in care, I didn’t realise how bad it was or is* (P10/D)  *One lady actually looks out the window every time I pull up outside and I can see her looking out the window and then she’s getting to the door to let me in because, as far as I can tell, that’s her focus, that’s her meal coming, do you know what I mean? That’s her one thing, one person, she is going to see all day? (P10/D)*  *It’s just a reliable, sort of, face turning up who has a bit of a chat who gives them a window on the world. We’ll talk about various subjects. A lot of it is fairly banal to be honest, but, you know, it’s just simply a bit of social interaction. it is the social interaction that a lot of them seem to value* (P11/D)  *I think the major effect is loneliness. It’s that isolation. Most of them live in quite a small world anyway. You know, they have either some, sort of, mental issues around dementia or they’re physically not as mobile as they used to be. So, very reliant on family or carers to do things for them* (P11/D)  *They’re just so grateful because a lot of these people might not even be able to get to see their family* (P12/D)  *Some people don't have anybody to speak to, and having us go in, it is not just a meal. It is about having that interaction with people, and that is certainly something I have seen whilst I have been going around* (P13/O)  *Some of them, to be honest they just love the interaction. I mentioned before, we get our drivers going in. They are seeing them every day, and that makes an impact for them. We find some service users just give our customer service team a call here, just to chat for 10, 15 minutes, (Laughter) just to have that, and the customer service team, [staff member] especially, she is someone who is superb at doing this, that will just call them and just have a chat, or they call us and they will just have a chat. Maybe a couple of things to do with menu changes, but you know they are really just calling to speak to us. So when we got in this morning, (Laughter) I could just hear [staff member] talking for about 15, 20 minutes to this person. So there is that sense that yes, some people are unable to cook for themselves, but some people also just want that human-to-human interaction on a daily basis, because we do need it* (P13/O)  *It is the same with the neighbours as well. We get neighbours calling us worried about their neighbours, if they don't think that they have anybody around them. We had one guy who literally has no family. It was just his next-door neighbour looking out for him and saying, "Look, during lockdown he is going to really struggle because we can't go in and see him. He is self-isolating", so then we get in touch, we help set up meals, and keep giving him that human interaction* (P13/O)  *Erm, I mean, I think that there's lots of elderly, vulnerable people and I think that they need a lot more, but I don't think that's really the department of meals on wheels. But I think that there's a lot of them that are without and are really lonely and need a lot more support than they get. But I'm not sure if that is the department of meals on wheels* (P15/D)  *So one I saw today, he's basically trapped in his house. He has dementia, so it's not good for him to walk around by himself, obviously, in case he can't remember how to get back. But he is not seeing anyone and is on his own a lot and is quite scared of the outside world. So a lot of them, if their families aren't able to visit them, it's just quite a sad existence sometimes* (P15/D)  *To be honest with you, I think a lot of it is they see somebody. If you don’t go in, they might not see anyone for days and days. Even though you’re not there very long, you might go in to one and she’ll say, “Have you got time for a cup of tea?” and it’s like, “No, I’m really sorry, I haven’t.” But it’s their way of trying to just get you to stay that little bit longer with them. I think a lot of it is the social aspect as well as receiving the meals, as well as receiving the wellbeing checks* (P17/D)  *The clients we go to, most of them, I would say probably, really look forward to somebody calling. I think it's, sort of, a marker for the middle of the day. It's somebody to chat to* (P18/D)  *Somebody going in there. Actually seeing a face, seeing a person. That is the biggest one, because they get very lonely. Mental wellbeing is a big one. Getting a hot meal. Someone checking on them* (P1/D)  *It’s just getting to know that person and making a difference to their day because some of them don’t see anybody. I think it’s seeing somebody and having that chat* (P2/D)  *You're just the first point of contact to them, or sometimes the only contact they have. They look forward to seeing you. They do look forward to you coming. They say it's a vital service to them. We think it is too* (P3/D)  *I’ve just been on holiday for two weeks and, having gone back, they’re all ever so pleased to see me* (P4/D)  *You can’t just walk out and leave somebody who’s crying or confused. I’ve got a lady who’s very confused and is often in bed. That’s actually another quite common thing. We will quite often find our clients in bed at the wrong time of day, particularly those with dementia. But not always, because some can be a bit depressed, as well. You know, living on their own. So, that’s something as well* (P4/D)  *Yes, the only people that will regularly turn up, is what I would say. So, if they’ve booked meals five days a week, then yes, we're the… They don't have care in place, then yes, we would be, probably, the only people that they see* (P5/O)  *I think, as well, because you're bringing in a friendly smile and a little bit of conversation, if they're stuck in a rut, and maybe quite angry that day, it changes the theme for them a little bit* (P5/O)  *When I get a phone call from a customer that’s got dementia, it can be quite a lengthy phone call. But I do think it kind of benefits, because they just get so much out of it, because most of the time they don’t have anybody to speak to* (P7/O)  *They are lonely and they do just want to chat sometimes. And, like I said, I haven't got the heart to say, “I’m really busy, I can’t speak now.” So I do, I do try and spend a bit of time, just to have a little chat about how they're feeling. I think it goes a long way* (P7/O)  *They do enjoy, as well, the driver, if there’s somebody on their own, they enjoy the driver going in for a quick chat and it breaks their day up as well* (P9/O)  *I can go into some of the houses that I deliver to and I’ve got to go round calling their name out because they might be just laid in bed or they might be just laid on the sofa, looking at a wall* (P10/D)  *Having somebody come to see them and a hot meal, just knowing there’re people out there who care* (P12/D)  *I've had customers say that, you know, I'm the only person they've spoken to and they're really grateful that I'm there…* (P15/D)  *It’s a face that they see that day because, sometimes, they don’t see anybody otherwise* (P16/O)  *You are walking in and you know when you close that door, they are not going to speak to anybody again and they are just going to be sat in that same chair until tomorrow in silence* (P13/O)  *They are waiting for you… I don’t think it’s necessarily for their food. It’s just they’re used to you going in at a particular time so if you’re late then they worry that you’re not going, which is quite sad as well* (P17/D)  *We had a lady yesterday who tried calling us. She left us five voicemails in the evening, and I think she just… her husband is in hospital at the moment and she just wanted that person to speak to* (P13/MO)  *[A client} said it’s not just about the dinner, it’s about us going in with a smile and having a chat that makes her feel so much better because she doesn’t see anybody. I think that’s a big difference* (P2/D)  *So the customer service team were signposting that, so we would always check first, "Are you looking for a hot meal?" If they are not looking for a hot meal, then we would signpost them onto other services* (P13/O)  *Sometimes, I think the idea of having meals is just that first step about just allowing someone to help you. Often, what we find is that we're delivering meals and then they'll open up to the idea: “Actually, this is quite nice, having someone come in.” So, it can lead on to better things* (P5/O)  *I think it's a step in the right direction. It's people that just don't feel they need help and so it's just that first rung on the ladder then, if you like, of giving in to having some support* (P5/O)  *I said to him, “I work in a day centre”, I said, “When the COVID is finished, get in contact with Care Direct, get in contact with Social Work, get your mum reviewed by a social worker and see if you can get her allocated – if it’s private, he might not be able to afford it but if he’s going through a social worker they might be able to get him funding to go to one of our day centres… he didn’t even know about it* (P10/D)  *We have been collaborating with Age UK and various other charities where we hand out leaflets signposting them to exercise classes, to various support- There is something called the support hub…* (P13/O)  **- Promoting independence and rehabilitation following hospital discharge**  *I think, a bit of it is because they want to be independent. I think also family are quite keen for them to be independent, as much as they can be* (P14/O)  *You do see people beginning to improve and feeling better about themselves because their house is a bit tidier. You know, they obviously just lost the ability at some point to take care of themselves and their house* (P4/D)  *We do offer a grocery pack for anybody that’s having a hospital discharge. So, on their first delivery, if it’s all put in place, say they wanted some teabags and some coffee and some ham and some cheese and some butter, or whatever, then we put a little grocery pack together for them with what they want and deliver that with their first meal* (P9/O)  *When they’re a hospital discharge, we do a home pack for them, which could be just the basics like bread, milk, sugar, things like that. And so, a few of our customers continue that, they continue having bread and milk delivered* (P16/O)  *But it can include people with mental health who are younger, or anyone that's struggling, so someone, for instance, who might have broken their leg and needs a bit of help. If they don't have family around, then we would help them* (P5/O)  *Then sometimes it will be next of kin ring up saying, “Oh, my mum is coming out of hospital next week, can I arrange the Meals Service for them?” So, we sort it all out for them and point them in the right direction* (P9/O)  *They can be a hospital discharge and they could just need the meals temporarily until they get back on their feet, in recovery* (P16/O)  *Sometimes going into residential care, it is not always the most pleasant of environments to go into. You are not in the safety of your own home, and certainly when I am going around. I think one, people, you can see they know their house, it is their own security* (P13/O)  *Well, without fail, I would say. Every time, because the budgets are so tight and because I don’t know why, government will have not given them as much money. Who knows. It's not my thing, but always, it's a question of justifying our existence. Some of the people making the decisions maybe have no idea of what we do and the benefits. The whole point of meals, or one of the points of Meals on Wheels is keeping old people in their homes. If you withdraw all services or price them out of people's range, then it's such a backwards step. They're all going to be in private homes, old people's homes and not in their own residential homes, which is crazy* (P18/D)  *Well, I think they are able to stay in their homes. If they couldn't have a hot meal or they didn't have somebody checking on them, maybe they would need to be in care, some people* (P3/D)  *Just the main thing is just that I feel that the service is vital to the community and to those who receive the meals. Because if they didn't get that and they didn't have that help, I just wonder where some of them would be. They would either be dead or they would need to be in care homes* (P3/D)  *We are dealing a lot with people almost on the edge of going into residential care or staying within their own home. It is the type of service users we have, whether that is with dementia- They are on that very edge, and we are that driving force behind to try and help them stay independent in their own homes whilst they still can* (P13/O) |
| **Benefits to employees** | **- Sense of pride, giving something back, being caring**  *It does give you a sense of satisfaction when you can help people out and although I’m in the office now, I still feel as though I’m helping people by giving them the Meals Service and hopefully giving them their choices that they want on the Service* (P9/O)  *But I love it, I love the job, I love speaking to the customers* (P7/O)  *As a vital member of the frontline team, I can see the value of not only supplying a hot meal every day but observing, helping and reporting back any issues that our service users have to the correct people such as social workers, family, through a dedicated office team* (P10/D)  *It was after losing my mother, I just felt that I wanted to work and be able to help* (P3/D)  *It’s just getting to know that person and making a difference to their day because some of them don’t see anybody* (P2/D)  *I just think I do like my job just because I know that it's really useful and I think it is a useful service* (P15/D)  *I do enjoy helping people and get a great satisfaction* (P10/D)  *… a lot of them [the drivers] can maybe be a bit shy about what they do. They go above and beyond every single time, and it constantly surprises me. We had someone whose birthday- He was 100 a few weeks ago, and they went and they gave them cake and a card. It is like you didn't have to, but it was during lockdown* (P13/O)  *When I get a phone call from a customer that’s got dementia, it can be quite a lengthy phone call. But I do think it kind of benefits, because they just get so much out of it, because most of the time they don’t have anybody to speak to. So, that’s really rewarding* (P7/O)  *We genuinely care about our customers and their wellbeing. So, yes, it does mean a lot to me* (P7/O)  *A lot of them [drivers] do very much care about this work, and it is not just a way to get money. A lot of them do take it quite personally, this job* (P13/O)  *I do find that really rewarding. When you go home and you know potentially that you’ve really helped someone, that’s a nice feeling* (P7/O)  *With fantastic team support I was soon able to do my bit for the community, delivering hot meals to the most vulnerable in our society* (P10/D)  *It's just nice seeing the people. Seeing them and being able to help them. Just being able to help really, to be honest with you* (P3/D)  *It’s generally a really sociable job. Just popping to see somebody and feeling that you might be the only person that they see all day* (P4/D)  *I think it’s a really, really, good service because we’re not care workers as such but we are caring people* (P12/D)  *… if it was simply delivering food and leaving, it probably wouldn’t have been as appealing. It was made clear that we offer a support role to the people we deliver to over and above just delivering the food* (P10/D)  *Yes, it’s nice to, sort of, give a bit back to the community really. I don’t do it for the money, believe me. It’s just a nice thing to do* (P6/D)  **- Reciprocal positive relationships with clients**  *The interactions I just find very interesting, very humbling. And you learn a lot* (P1/D)  *I love it. I absolutely love it, yes. It’s surprising how well you can get to know someone just delivering a dinner* (P2/D)  *I‘ve been delivering to some of the people for 10 years plus. My children have grown up, they know who my children are just by me telling them* (P2/D)  *I know a lot of the clients by their first name and they know my first name, and so I try and make it, not formal, but a little bit personal to them* (P6/D)  *They became a bit like my family, I knew their life stories and I felt like they knew mine* (P12/D)  *That first time you walk in that house you click. For whatever reason, you just click. Then the nature of the job is that person passes away. Then you find yourself going to funerals and stuff like that. Then you meet families, and they say, “Who are you?” “I am P1Driver. I did Meals on Wheels.” “Oh, how lovely to meet you,” blah, blah, blah. Yes, I have done my share of funerals over the years* (P1/D)  *We’ve had a couple of them where they have reached the grand old age of 100 or it’s just a significant birthday as they get older. I’ve said to my boss, “Can I take an extra cake?” Obviously we check, their dietary requirements, they’re allowed cake, because obviously some people aren’t. You have to check that, you can’t just, “Oh, have a piece of cake.” Then make them ill. So things like that. My boss is always saying, “You go out of your way for that.” I do, because I try to think… I try to treat people how I would like to be treated. I’ve always been like that in any line of job I’ve done. It’s just that extra little bit of personal touch, you know* (P12/D)  *I’m on first-name terms. I’ve only been doing it a few months, and I’m on first-name terms with nearly all of the clients already, and I think it’s just that, sort of… how can I best put it? It’s just a reliable, sort of, face turning up who has a bit of a chat who gives them a window on the world. We’ll talk about various subjects. A lot of it is fairly banal to be honest, but, you know, it’s just simply a bit of social interaction* (P11/D)  *Oh, it is brilliant, listening to the stories, because you have only got a limited time when you go in somewhere. So going in there every day you get a little bit, and then a little bit more, and then a little bit more. It is lovely* (P1/D)  *I think to some of them it is therapeutic, because it is going round their head all the time. Some of them will just have a little mention because they have been thinking about it that day, when they have their D-Day celebrations and stuff like that, the Cenotaph and Remembrance Sunday. That gets very emotional* (P1/D)  *They worry about you because you haven’t been seen. Some of them are just really happy to see you, which is really nice* (P17/D)  *An important perceived benefit revolved around life experiences, sharing stories and learning from clients.I had a lady on my round. She had an answer for everything. She used to say, “Everything alright?” And I would say, “Oh, yes,” blah, blah, blah, “but this is going on in my life.” And she said, “Well, what you do… This is how you do this. That is how you deal with that.” So it is not just us helping them. On the odd occasion they help us. Life experience. You can’t beat it* (P1/D)  *I enjoy seeing my clients every day. You build up a really nice relationship over the years because some of them I’ve been delivering to for many, many years* (P4/D)  *… it is person-centred. So, I spend a lot of time talking to the people I deliver to* (P11/D)  *Just the interaction, the feeling like you’re worth something, the appreciation. Yes, 9 times out of 10, they really appreciate you* (P12/D) |
| **Being the fourth emergency service** | **- Being the first responders**  *With this one client that we had, one of the drivers went in last Wednesday, and this guy was collapsed on the floor covered in his own faeces. He couldn't move. He had been trying to get up the entire time and had scraped his whole back. He was a very thin man anyway to start with, but literally was dehydrated. His heating was on full because he hadn't been able to turn it off all night* (P13/O)  *I went in one lady and she has got dementia and I was serving her, putting her meal up, and she said to me that - she was stood by the sofa and she was holding on to the sofa and she said, “Oh, I’m hurting.” I said, “What’s the matter?” She said, “My hip hurts.” They rang the family, the family went round and she had broken her hip. So, they phoned… they had e-mailed the office to say ‘Thank you very much for looking after her’, you know, they may not have picked up that she’d broken her hip if we hadn’t have been there. She’d have suffered all day* (P10/D)  *I had another lady I was delivering to. I turned up there one day and she was walking along wincing in pain. I said, “What on earth have you done?” She said, “I don’t know, I’ve hurt my arm.” As it turned out I got an ambulance out and she’d dislocated her shoulder* (P2/D)  *I had a lady this morning who had obviously fallen over. She’s had no personal assistant today. When I went in, she was, sort of, half on the floor and half on, like, a sofa, and, obviously, I’ve got to deal with that. So, I had to get that sorted out. I phoned in the office and asked if they could phone her relatives, told her the situation, because I don’t phone the relatives to check on her. Between us, we got her back onto the settee, etc., but I’ve got to be careful, and then I phoned in work. They said they’d pass the message on and somebody would be coming to see her today, to make sure she was okay* (P6/D)  *I've had customers who have, for example, fallen and I've been able to either get an ambulance or, in a care home, get the care home manager. So maybe they would be there for a long time on the floor if we hadn't been there* (P15/D)  *Sometimes you go in and somebody is very confused and really not themselves. Often, it's something simple, like a water infection. So, that would be definitely a case of not phoning in at the end of the day. That would be a question of phoning then and there to the office to advise them that Mrs so and so is definitely not herself and is very confused and disorientated and just not with it at all. So, you would phone the office and they would either, dependent on what next of kin arrangements there were, either phone next of kin or get in touch with the GP* (P18/D)  *One of the challenges is if you find somebody that’s dead. I went into a lady’s house about a month ago and I knocked her door and she always answers the door straight away. I knocked two or three times, looked at my sheet and it said knock the neighbour across the road which I did. We’d gone in and she was just sat there. She’d been sick. She looked dead. That sounds quite awful but she did really look dead. I thought she’d had a stroke. I phoned an ambulance. Her daughter did arrive within 10 minutes* (P17/D)  *To have that mindset, that if you pass away someone might not… If we weren’t going in and they didn’t see anybody else, they could be in that house for a week or so. And that is quite scary to some people (P1/D)*  *I have found everything over the years. I don’t think there is anything I haven’t found. Heart attacks. Full strokes, where they are on the floor, non-responsive. Three dead ones in sixteen years* (P1/D)  *If they haven’t got smoke alarms, we refer them and the fire brigade go out for free, which is really good* (P2/D)  *I remember going into her house one day and she was cooking a gone off chicken in the microwave. It was vile. I had to ring the office to get them to sort it out because I wasn’t allowed to touch it* (P2/D)  *But it does happen that you will go into a client’s house and find that they’ve fallen and they’ve been on the floor quite a long time. So, that’s quite hard. I don’t know how many times it’s happened to me. I mean, it’s not regularly. I would say once every six months or so. And sometimes, it’s a simple matter of calling an ambulance or some district nurses might come out and help them up and you have to wait for the ambulance people to come, obviously, and sit with them and make sure the office is calling their family and that sort of thing* (P4/D)  *And sometimes, unfortunately, our drivers go in and that person has passed away, and no-one knows, and then our driver has to feed that back, which is not nice. But again, they sit with them. They sit and they talk to them and they wait for an ambulance to come out. You know, it’s just- Yes, I just think it’s- Like I said at the beginning of this, it’s way more, way more, than just a meal service* (P7/O)  *And another lady, I'd been marking down that I felt like she was deteriorating quite a lot, and I found her on the floor one day. And she said she'd bumped her head. So I phoned the ambulance for that one* (P15/D)  *I went in there and she was sat there. She was speaking but her eyes weren’t quite right. Her speech was slightly slurred, and she was having a mini stroke…I rang up the office, who rang an ambulance for me, and I waited for the ambulance to get there and they took her in* (P1/D)  *And another lady, I'd been marking down that I felt like she was deteriorating quite a lot, and I found her on the floor one day. And she said she'd bumped her head. So I phoned the ambulance for that one* (P15/D)  *Of course, the other side of it, we have gone in where customers have been collapsed on the floor. Back in February this year we had one of our drivers walked in, a lady in her 80s had been assaulted. She was passed out on the floor, like stone cold, battered and bruised, her place had been completely robbed. Our driver was the first to go in there, and she had no other carers who would have gone in to check on her. It is only because we were going in that then we were able to raise this* (P13/O)  *If customers do feel like they’re not coping, or whatever, then we report that to our office and our office would then go to people like Care Direct. Care Direct can perhaps arrange external visits. You know, like, support for company. You know, like, they were doing, or food packages, things like that. So, hopefully, there would be something in place for those customers, that way* (P16/O)  *We’ve had instances where people have had to call ambulances and they had to stay with the client while the ambulance arrived* (P10/D)  *We’re trained to get in touch with the office because the office have all the next of kin details. Then they would take that to the next stage, trying to get hold of the next of kin. We do have a telephone number for them, but they might have another number* (P12/D)  *Yes, we do well-being checks, but for some people we are literally their lifeline by going in and making sure- She was collapsed on the floor. We were then able to call the police, the ambulance services. I mean if you were to go through the customer calls that we get coming into the service team here every week, there are, I would say, at least one or two ambulance calls going on and where our drivers are just sat there, staying with the service user until the ambulance crews come, or maybe a member of the family comes to take over, to make sure that they are okay* (P13/O)  *The office staff will always ring the customer and if we don’t get any answer then we would contact family, if they have it. If not we would contact, sort of like Care Direct. If it’s on a weekend we would check hospitals, because we’re a bit more limited on weekend. Obviously, there’s no other council services available on a weekend, so our only option is, if we can’t get hold of family or whatever, is to leave a message for them. But also just to contact hospitals, to check to see if they’ve been admitted* (P14/O)  *Basically, they’ve always got support at the office, so if they feel they needed to ring an ambulance for anything, then they’d report to the office to say that they’d called an ambulance. They are always in contact with the office, so if they have any problems, they can just ring the office and we can either talk them through any problems or what they need to do* (P16/O)  *To be quite honest with you, I think then they would contact Adult Social Services and get them involved. I’m pretty sure that’s the protocol of how it would go* (P17/D)  *You're there, you're on site, you make the call whether you need to call an ambulance, whether you need to call the office to let them know what is happening* (P3/D)  *Yes. They go to business support and business support go to the social worker. If there is no social worker then they do a safeguarding, they go to that. I’m assuming that’s the social worker anyway. They create a safeguarding issue…* (P2/D)  *Generally, when we are asked to come in on these cases, then Social Services will be getting involved and they will start to have people to help them with looking after their house and clearing out and that sort of thing* (P4/D)  *We have in the past had the community police come out with us, so because they were always keen to know who the vulnerable people were on their patch* (P5/O)  *The other thing, as well, we can call upon other services within [the area]. Like occupational therapists, we encourage them to come out and help us* (P5/O)  *I like being able to solve issues, if you like. If a customer is unwell, which does happen quite often, I get in touch with their family, so we’re able to kind of feed that on, so that person is not left to deteriorate. And then obviously the worst outcome- So, we’re kind of then- We can step in straight away and raise the alarm, if you like, to get that person the help that they need* (P7/O)  *[If the client does not answer the door, the driver may use a key safe, if the client is not home and is nowhere to be seen, then:] The customer service advisers then would first of all try and contact the service user on their mobile or their telephone. If that is not successful, then it goes to the next of kin. If that is not successful or they don't have any next of kin, then they start calling round to the hospital, various hospitals within the council. If they are someone who receives a support package, we have an internal management system called LAS, where they would raise it there with a social worker or they would add it to the profile, yes, that they didn't answer* (P13/O)  *[If the client was not at home and could not be found] So you would then have to ring around all the hospitals, and find out if they’d been admitted.The majority of the time, I’m able to be put through to the ward that that client is on and I speak to the staff nurse, and just explain obviously, “This is a customer of ours, they’ve got no next of kin that we’re aware of, they really rely on these meals. So, we don’t want them being discharged without any notice, because they won't be getting meals. Until you let us know that they're home, they will just be suspended.” So, I do try and stress that, “It’s really important that you let us know, so that then we can bring a meal the day that she’s coming home,” essentially* (P7/O)  *And if she was referred by the social worker in the beginning, then we could always ring the social worker and find out the issues with the social worker. So they could then inform the care agency, or etc., etc* (P7/O)  *We take it upon ourselves to have that kind of relationship, especially with hospitals* (P7/O)  *One of our drivers went out in his round to a regular customer of his. He went into her house and found her in bed, which is not like her at all, she’s always up, she’s dressed, she’s talkative. She was completely unresponsive, covered in vomit, really, really unwell. And he just rang 999 straight away. He stayed with her, held her hand until the ambulance got there, and potentially saved her life, because she’d had a massive stroke. And the family just- As you can imagine. I took the call on the weekend and they were in tears, saying, “If it wasn't for your driver, we wouldn’t have our mum now.” Because no-one was going to go in all weekend, she doesn’t have carers, no-one would have known. So that, I think, speaks volumes for our service* (P7/O)  *I know a bit about first aid and I have seen stroke victims. So, I know what I’m looking for in that way, about the mouth down and stuff like that and the arm, but if I’ve seen anything like that, then I would have phone 999 straightaway and rang the office* (P8/D)  *Sometimes they come through Care Services which is – we’ve got a LAS system, which is Liquidlogic system. What that is, that’s connected, the hospitals and the Care Services use that system. So, they can refer somebody through on that* (P9/O)  *Recently we had somebody that had fallen down and we had to get an ambulance for them. The place was in more than a mess. So, we had to get Care Services in to clean up. I mean, in all fairness, I had to hand it to my line manager, in the end, because nobody was, basically, listening to me. So, yes, they had to step in as well because the hospital wanted to send him home and the place hadn’t been cleaned up* (P9/O)  *We can find someone on the floor and we stay there until we get help either from someone to just come or sit waiting for an ambulance. It’s not just about the dinner, it’s about everything else that we do* (P2/D)  *If they haven’t got smoke alarms, we refer them and the fire brigade go out for free, which is really good* (P2/D)  *I found a lady who had been attacked in her own house. Originally, I thought she’d had a stroke because of the way she was talking to me and the way she was. As it became more apparent, and the family turned up, somebody had actually broken into her house. Obviously, that was all reported through to the police* (P12/D)  *Where we’ve supported and waited for ambulances to arrive and all these types of things have happened, where the staff stay with the customer, until appropriate help is at hand* (P11/D)  *I’m able to report that back to my bosses and then… they report back to the social worker, they can then contact the carers, the carers can go in and assess the situation. So, it’s like teamwork, like being collaborative. It’s like making sure you communicate with other people so that everybody knows the situation* (P10/D)  **- Getting food to clients during adverse circumstances**  *I think COVID has highlighted that more than, possibly, the snow because people have had to isolate and we might have been the only people going to see them, apart from their immediate carers* (P12/D)  *Come weather, come illness, come epidemic, whatever it is, we’ll still do our very best* (P12/D)  *I think that is the whole crux of the matter. We have got to keep doing what we are doing* (P16/O)  *Yes, they know they’re going to get a hot meal, regardless of anything* (P6/D)  *That can be a bit of a challenge getting the meals out but we haven’t missed anybody yet* (P9/O)  *Hopefully, this service will carry on throughout, and be highlighted to know that we are here for the help out there, for anybody that needs it* (P16/O)  *It's a frontline service that we make sure we get out there every day* (P5/O)  *So, I mean the meal service definitely is an essential service* (P8/D)  *Because… also with everything which is now going on with COVID, we’re a lifeline to a lot of these people because they might not really see anybody else* (P12/D)  *… come rain, sun, ice, snow, it doesn’t matter, they will get that meal. We’ll deliver meals regardless* (P6/D) |
| **Wider benefits to the community** | **- Reducing pressures on families**  *A lot of families work full-time. They just can’t get there during the day, can’t get to cook regularly for them. They just haven’t got the time, really* (P4/D)  *So the families like to use us, so it takes a little workload off them. They know that someone is coming in, checking on them, giving them a meal, so that they don’t have to worry constantly about them* (P1/D)  *I went into one house and his mother has got dementia and he is in the kitchen and he’s almost in tears. I said, “Are you okay?” He said, “No, not really.” He said, “I had a really bad day yesterday with my mum.” He said, “She was really bad, she was shouting, and I don’t know what to do.” Because it’s not just about the people that are ill, it’s about the people that care for them, as well. The bloke was at his wit’s end. Do you know what, he actually thanked me the next day for actually just spending the time to talk to him* (P10/D)  *Again, I think a relative thing to think it’s more where we can keep an eye on them and make sure that they’re okay so the relatives don’t have to go in all the time* (P17/D)  *A lot of families aren’t local anymore. You know, a lot of families are very spread all over the place, so that’s another thing* (P4/D)  *Obviously the customers don’t really realise that we’re doing wellbeing checks as we’re going in but obviously the relatives do. Sometimes that’s enough just to keep them home without having to go into a home* (P17/D)  *Often, it is at the family's request that we start going. They are concerned that their elderly parent or whatever is not looking after themselves as well as they might. The family like it because it's a check to see that they're okay. We obviously observe them daily, so get to know when things aren't quite right. So, definitely it's beneficial, for all sorts of reasons, but mainly that* (P18/D)  *The family were so grateful. When I went and gave my statement to the police, he was like, “You know what, if you hadn’t got there within the hour she probably would’ve died.”* (P12/D)  *Even when people, sadly, die, we’ve had many a card and a letter saying, “Thank you for delivering to my mum or my dad, or whoever it might be. You’ve made such a difference.” they’re just so grateful because a lot of these people might not even be able to get to see their family* (P12/D)  *Family call us and say, "We want to start paying for meals for our mum", or dad, "We don't think they can do it themselves anymore." Then, of course, we would then give a phone call to the mum and dad and go through the menu options and explain what the service is about. So you also get some families for more reassurance for themselves, that they have somebody going in looking after their loved ones* (P13/O)  *That’s it, because they know if there is a problem. If we’ve got any concerns at all then we’ll be straight on the phone to them, telling them we’ve noticed a problem* (P14/O)  *Obviously, then, if it were more serious, that they’d had a fall and an ambulance had been called, it’s keeping that family informed of what is going on. So, at least, then, they would know that they’d at least been taken to hospital. So, that’s the family’s benefit side* (P16/O)  *Because sometimes when family live away, especially when they weren't able to come and visit, a couple of times we had phone calls from, say, their daughter, and passed on by the office to say… one was, "Can you have a look because mother's lost her teeth." So, then you're looking around the apartment or flat or whatever. Or, "Can you check on this?" Or, "Has she got that?" All sorts of things, that because you're allowed to go in and visit them, you can get back to the family and put their minds at rest* (P18/D)  *There are benefits to their family, for certain. If you're going to visit… it helps the carer, if you like* (P5/O)  *So, if we can ring that family member and say, “We’ve plated their meal, we’ve cut it up for them, we’ve put it in front of them, put the TV on for them, they're sat there eating their meal,” it’s an amazing relief for their son or their daughter, to know they're eating* (P7/O)  *I mean, people… especially next of kins of the customers have said they don’t know what they’d do without us* (P9/O)  *Obviously, relatives, if they’re working then they’ve got their own lives, you know. They can’t come over at lunchtime and cook mum a dinner or whatever. So at least if they know we’re going in, it gives them peace of mind* (P14/O)  *The other side of it as well is reassurance for family members, and particularly during COVID-19. You have got families who are living over in London, living over in Wales, living all across the country, and they can't get down to go and see their families. They know that their parents struggle to have their food* (P13/O)  *For the family, I suppose it’s the wellbeing check thing, as it’s somebody who is going in and checking on their parents, or their family members, and ensuring that everything is okay. Obviously, if there was a problem, we report anything back to the family, that’s who we would go to first* (P16/O)  *I think the families may be attracted by that [the welfare checks] because they’re aware that when there is an issue, we, as drivers, if it’s an immediate problem, we stay with the customer and wait until help arrives if it’s required, or if it’s not so much, we report back to the office who then interact or talk to a member of the family to report a concern that we’ve raised regarding the parent or family member* (P11/D)  *You’re providing a service for that person and for their family* (P10/D)  *It is an absolute lifeline. We hear it a lot from families, that, if they didn’t have that, they wouldn’t be able to live at home. So, I think it is providing such a great relief for families as well* (P7/O)  *That’s what the families get out of it is the wellbeing check and they know that when they’re at work there’s somebody calling in on their elderly relatives and they’re being looked after. They do know that if there’s any problems during that day, they’ll get a phone call from us* (P9/O) |
| **Challenges faced by the service** | **- Organisational challenges**  *Funding and because we’ve always kind of been the forgotten service. We’ve always been the service in a way that’s been under threat of being got rid of, because nobody saw the point of us. So we were never really given support and funding that, you know, would have made it a bit easier. I think definitely that has all changed in this last year* (P14/O)  *Our service has been under threat of closure quite a lot over past years. But we’ve still carried on* (P16/O)  *… we’ve always been the bottom of the pile, so to speak. Which I think has always been sad, because they just didn’t understand what kind of service it was, you know* (P14/O)  *I mean, in addition, the cost of the food itself, they have to fund us as drivers and the vehicles and whatever. So, there has to be increased funding. It’s an enhanced service. You know, we’re not a commercial enterprise. They have to find the value, the fact that we deliver the extra service alongside the food* (P11/D)  *Yes, absolutely and it's short-sighted to think that it's something that you could perhaps cut back on and expect people still to be able to manage* (P18/D)  *Obviously, the challenge as well is keeping the service going. The dinners have gone up because the costs of everything go up. It’s keeping your numbers up as well, so the council don’t cut the service. As much as I don’t have to deal with that, that’s more management that has deal with that. I’d say the cost of the meals actually would be our biggest challenge really* (P2/D)  *There is always, every year, "Will it continue?" "Will the council find the money?" "Will they price everybody out?"… "Will they make the meals so expensive that nobody's going to have them?". All of those budget concerns and whether the councils generally think that it is a viable thing to keep going* (P18/D)  *Then we had an integrated office and driver. We were all employed by [name of Council], comparatively recently. I don't know how many years now. They have outsourced it. So, now it's [name of private company]. I mean, I get on fine with the people I talk to. I do still feel that there is a slight disconnect because we're not all under the one flagship. But hey ho, that's progress, isn't it?* (P18/D)  *No. A lot of them are – I am going to sound old now – young kids in their early 20s, mid-20s, maybe 30s. They haven’t got any experience in any of this whatsoever. They are basically just looking at a computer screen. They don’t always give the sheets out to do. You have to ask for them. But again that is just because our office is not being run by [name of Council]. It is being run by [name of private company]* (P18/D)  *A lot of them are – I am going to sound old now – young kids in their early 20s, mid-20s, maybe 30s. They haven’t got any experience in any of this whatsoever. They are basically just looking at a computer screen* (P18/D)  *No disrespect to young people at all, but some of them don't have the life skills… That sounds awful, doesn't it? But just something… Yes, some things are serious and need to be taken serious, and they may not be… I always feel in my job that I'm, kind of, looking over their shoulders a little bit and making sure things are happening, so it becomes a little bit negative. I don't want to be like that. I want to be encouraged that things are being looked at properly, and being dealt with properly and followed through properly* (P5/O)  *When [name of Council] used to run our own office, our office was brilliant. Now it has been taken over, they have outsourced to [name of company], it is not so good. I used to be able to ring in, and I would say who the service user was and our office would know who they were, everything. Now, the people in the office are not really… it is just a job. They are just office. It is not so good* (P1/D)  *I mean people used to say to us, “Oh, I didn’t think you were still going. I thought you got closed down years ago.” Because it was never advertised or whatever, people just didn’t know we existed, they thought we’d gone* (P14/O)  *Obviously, the service is very much word of mouth as well. We’ve talked about how we can advertise the service more* (P12/D)  *They were talking about, only a few weeks ago, “How can we broadcast the service more?” I said, “Would it be an idea to have one of the vans, not all tarted up but some sort of Meals on Wheels service going in and around Bristol.” You know, it doesn’t have to be every single van just help us publicise the service if at all possible because I think it is an amazing service and we work really hard to deliver it* (P12/D)  *Looking at how we advertise the service, should we be looking more to social media? I mean we have only just started now really setting up posters and leaflets in various community centres and council buildings about the service. I mean prior to that, it was more just word of mouth.*  *You often get that people are surprised. "They are still a meals service" often comes up, but I think the direction we are going in, the big focus, is making it as accessible as possible and to get us known within our own council, (Laughter) that there is this service available. So we have done things like- We call it 'The Extra Mile', which is a way of the council recognising parts of the organisation that have gone above and beyond. So we featured on there, we featured on adult social care briefings across the council, and looking at our statistics, both from private customers and customers referred through from our social services, it has all seen an increase. So our exposure is getting bigger, but there is much more to do to make it more accessible* (P13/O)  *We’re sort of promoting more and we are putting on them more about the welfare of the customer, and it not just being about a dinner* (P14/O)  *Hopefully, this service will carry on throughout, and be highlighted to know that we are here for the help out there, for anybody that needs it* (P16/O)  *We're trying to do promotional stuff a little bit different these days. Quite often, that's when the older adults, if they can get out, would go to the market. I would just approach them and say, “I'm not approaching you because I think you need Meals on Wheels, but are you aware that the service is still running?” “No, I didn't know that was still going. God, I thought that went years ago.” “No, it's still running.”* (P5/O)  *You would assume that it’s all older people, but it isn't, it’s young people that have maybe got a brain injury or drug and alcohol misuse. Things like that really, so it covers quite a wide range of ages. And I think a lot of people think, “Oh, they won’t be open on a weekend.” So, I mainly get it from family members, just ringing on the off-chance and then going, “Oh my God, you're open.”* (P7/O)  *When people say ‘Meals on Wheels’, they don’t quite understand or appreciate, I don’t think, the amount of work that goes into it* (P7/O)  *We’re doing advertising at the moment. [My manager] has, you know, arranged posters that are going out to clinics and old people’s homes and, like, community places* (P9/O)  *I don’t think people realise how important Meals on Wheels are. I really don’t think they do… that the general public really understand the intenseness of it* (P17D)  *Anybody can have Meals on Wheels. It is a misconception that they have to be old* (P1/D)  *We are trying to address… the image that is portrayed, because… it is often associated with the older clients. So we are trying to look at ways that we can make it more accessible* (P13/O)  *I, like a lot of people, didn’t realise this service still existed, and I didn’t also realise that part of the service was to offer a bit of support* (P11/D)  **- Restrictions on time spent with clients**  *Usually on a Thursday I’ve got about 24 deliveries. Friday I’ll have 30, plus I have to collect money, so, that all takes up time. So, what I do to prepare myself is, I write the receipts out while I’ve got that hour waiting for the food to cook. It gives me that extra few minutes that allows me to talk to somebody. Because they want you to stay there and talk* (P10/D)  *Especially if you're running out of time. And yes, you can't spend an afternoon talking to someone because then people wouldn't have any dinner* (P15/D)  *Unfortunately, if you go into a customer and they’ve got them on the floor, they have to wait there until either an ambulance is there or a family member can go there and take over, or anything like that. So, they could be held up for 20 minutes, half an hour, 45 minutes* (P16.O)  *Even though you’re not there very long, you might go in to one and she’ll say, “Have you got time for a cup of tea?” and it’s like, “No, I’m really sorry, I haven’t.” But it’s their way of trying to just get you to stay that little bit longer with them. I think a lot of it is the social aspect as well as receiving the meals, as well as receiving the wellbeing checks* (P17/D)  *You have to be a little bit more assertive and just say, “Look, I’m really sorry.” Not be rude to them but, “I really haven’t got time for this. I’m here to deliver your dinner. I still have 32 to deliver.”* (P17/D)  *Yes. You get this instinct. When you are talking to somebody you find yourself walking backwards as you are talking to them. You are working your way to the door as you are still engaging with them and talking to them. So yes, you do learn. You have got that timer in your head. It can be very sad, because you don’t want to leave somebody. That is a big challenge, yes, because you would want more time* (P1/D)  *Others you could be there up to 10 minutes. Generally, you want to do it about five minutes per one because obviously you’ve got to get the dinners out as well (P2/D)*  *But, of course, it varies from client to client. Some don’t want to chat. You just pop their lunch down for them and leave, just make sure they’re okay, and they’re happy with that. Some people like to have a little chat, so it, sort of, balances out, really. You can spend a few more minutes with one and not so long with another, really. And that’s a bit of a call, really, as to whether they want to get up. It’s very difficult, because you can’t rush somebody to do that sort of thing and, if you have to sit with them for 10-15 minutes, and you do, you know, you wouldn’t leave. You wouldn’t walk away* (P4/D)  *It’s always seemed to be a bit of a time constraint because, obviously, they like to have a chat* (P6/D)  *And we have to get it done by two o’clock. That’s the cut-off time. So, it’s a really fine line to try and spend time with people and divide it up equally. It’s a really hard thing to do. I think they’re aware of it. We’re like the unsung heroes, if you like. Nobody really knows what our job is until they have a go at it themselves, and it is a bit of an eye-opener* (P6/D)  *Trying to do all the drops that the drivers have on a weekend and meeting the deadline, they have to- Their finish time is 2:00pm and, more often than not, they don’t really finish on time. And then obviously the later the drivers come back, the later I go home, because I have to process all the paperwork, etc. So, it just has a bit of a knock-on effect. I think that’s what we all find a little bit difficult, we’d all hope there was more time, I guess* (P7/O)  *We’ve got a two-hour window to deliver the food at a time that’s probably acceptable to have lunch, and so with the volume of deliveries, we can’t really afford any more time than that, unless there’s an emergency, in which case we stop everything and attend to the emergency* (P11/D)  *But the management do listen to us when we say things like that. They do their best to re-route them a little bit and get the numbers back down for us. It can be challenging in that sense. You do think, “Oh goodness me,” especially if you are running late and then you’re later again. Then like I said to you, people are, “Oh God, you’re late.” You hear it continuously every house you go into. You can’t win because you’re either too early or too late. You can’t win. There can be challenges like that. It can be quite a stressful job. I think you really need to enjoy doing the work for it to work for you. If you really don’t enjoy going in and out of people’s houses, you see lots of different things. You see how they live* (P17/D)  *Probably no more than three to four minutes with each. If I’m plating, we’ll have a conversation as I do it…some are very, very chatty. You know, they would like you to sit down with them for a good 15 minutes, which we can’t do, obviously* (P11/D)  *Erm, maybe sometimes there's too many drops to do and not enough time. So, ideally, it would be nicer to spend more time with the people. But there's sometimes just not enough time* (P15/D)  *I am conscious that they may not get necessarily enough time with people* (P13/O)  *I mean they’ve only got like three minutes, but in those three minutes they have to take in so much… they’ve just got to be so observant and just check on everything* (P14/O)  *The job is very time restricted. You have to get 30 odd meals delivered in a specific time* (P10/D)  *As you are plating the meal up you are looking around. You are trying to do as many things at the same time, to save time, but to make sure that you are doing it* (P1/D)  *What I do to prepare myself is, I write the receipts out while I’ve got that hour waiting for the food to cook. It gives me that extra few minutes that allows me to talk to somebody* (P10/D)  *The advice I was given by the drivers is spend a bit of time with different characters each day, so that everyone gets a bit of time when you have an extended chat with them* (P11/D)  *Nothing major. Maybe 10-15 minutes. But that would make a hell of a difference* (P1/D) |
| **Challenges that emerged during the pandemic** | **- Demands on resources**  *During the second week of lockdown I was asked to relocate to the Meals Service as they were short on drivers and I had previous experience driving (*P10/D)  *I think the only biggest issue for me would be down to staffing* (P13/O)  *I mean, our numbers have increased with COVID-19. I think it brought home a lot the value of the service* (P16/O)  *Quite a challenge because you obviously get a shortage of staff. And was it one time when we were down 50% of staff? Well, 50% of drivers. And then, obviously, we’ve got a member of staff in the office who covers rounds sometimes, in emergencies. I cover rounds in emergencies. When we’re short-staffed or if I can’t get a round covered, I would go out and do deliveries. So, it’s been like that* (P16/O)  *We’ve recruited quite a few casuals recently and that’s been really good* (P4/D)  *It might be that one driver does three days and the other does two, and then each of them does every other weekend. Yes, so we've increased our staff, but we've got better cover* (P5/O)  *And because we’ve got three, or four actually, phone lines, they can all be going at once. So, in that respect, I do have to prioritise my workload, be careful with my time. I’m continuing to do my computer work while I’m talking to a customer on the phone, that’s absolutely fine. But when all the phone lines start going, I just say, “I have to go, I have to answer these other phone calls.” So, it is a bit of a juggling act* (P7/O)  *Coronavirus, we were having to do a waiting list, because we literally just couldn’t manage the influx of people all at once. So, there was a waiting list in place then* (P7/O)  *We’re short on the ground now. Sometimes one of my colleagues, or me, will go out and help drive, while somebody else delivers, if they don’t know the route* (P9/O)  *We carry PPE in all the vans, I wear a mask, we sanitise after every delivery through hand gel* (P11/D)  *Yes, no, we’ve never run out. I think there was just a tiny little bit with the hand sanitiser but then that was, kind of, nationwide. We were never left with anything not. We just sourced out different places to get it, do you know what I mean, from where we were getting it before* (P12/D)  *We didn’t have any PPE to start with. It was quite frightening, especially when you’ve got children at home and you’re out all day, then you’re coming home. Obviously you don’t want to pass anything to them* (P17/D)  *I think we’ve been very well prepared. We’ve had lots of PPE. We’ve got no shortage of that at all and I think we’ve all coped pretty well, actually* (P4/D)  *I was worried, when it all started. I didn’t want to go in, because of obviously all the drivers coming back from people’s homes. And I’ve got a little boy that’s not well and my mum is disabled, so I was really worried. But when I walked in there, everything they put into place straightaway was just amazing* (P7/O)  *… and the clipboards, and stuff that come back is all wiped over before it’s given to us. We’ve got sanitisers and the drivers use masks as well now. We don’t, in the office. If we went out to a customer, then we’d be wearing masks, and PPE. Yes, they’ve got their full PPE and they’ve got sanitiser in the vans as well now* (P9/O)  *I was working seven or eight days on the trot before having a day off and maybe just having one day off and then going back because the numbers went up* (P17/D)  *Not in the beginning. No, not in the beginning. One of our drivers works somewhere else and he was bringing us some gloves in. We didn’t have an awful lot, they struggled to get it. In fairness to them now we have an endless supply of it all. Handwash, gloves, masks, aprons, we’re very lucky* (P2/D)  *To be honest, I'm really missing it, but that's just one of the things – one of the things that we have to adapt to in life, isn't it? Because, yes, when you're driving around with a driver, you get to know the driver a little bit more, what makes them tick and all those sorts of things, and how they're dealing with different things, but just not getting that interaction at the minute* (P5/O)  *The uptake since coronavirus is massive (P7/O).As a result, demand for food also increased.I mean, my challenges would have been to do with getting the food in. So the company that we get our food from obviously having problems delivering* (P14/O)  *We got extra drivers in… because the day centres and everything closed down, there was excess staff there, drivers and catering staff, so we were able to use them. So that meant that we could increase the number of rounds that we had to cover all the extra people that wanted the dinners* (P14/O)  *We hired more vehicles. We increased our orders with our food suppliers* (P13/O)  *… a couple of my colleagues had to be out because their children were sick and they didn’t know whether they had contracted COVID* (P9/O)  *COVID has made it difficult because they [new drivers in training] haven’t been allowed to come in the cars with us. You have to take two cars* (P1/D)  *I would hope we will be better prepared this time. Because we didn’t have any PPE to start off* (P1/D)  *I think obviously with the PPE, we will make sure that we’ve got enough PPE for the staff, which was always hard to do at the beginning* (P14/O)  *We were very PPE’d up, if that’s the right word, at the beginning. We had all our gloves, our masks. We’re still wearing masks now. We’re still sanitising our hands all the time. None of that has really changed* (P12/D)  *So if they weren’t able to supply, then we were okay. I mean we’ve also got a contingency stock of about 200 meals, because we’re part of the Council’s emergency planning… so if there was a major disaster then we would be called upon to supply meals. So we always have to make sure we’ve got meals* (P14/O)  *I think we are quite well-rehearsed now using PPE. That is not something to be scared of, that we are scared of anymore. We are well-stocked with PPE* (P13/O)  *We have seen another jump in customers just this week as well. We have lots more people calling up with queries, trying to find out more about the service and what we have to offer* (P13/O)  *It was bad because, you’ve got to imagine, when [the pandemic] all hit, a lot of their drivers went off sick, obviously for good reasons, and they were desperate for drivers* (P10/D)    **- Impact on client and staff wellbeing**  *Then you use PPE and keep yourself as far away from them, if you can, as possible. But again, like I said, with people with dementia, they don’t really understand. Yes, because some of them would be like, “Oh my goodness.” Now they’re all quite used to us walking in with masks on so they don’t take any notice at all. But yes, at first it was quite daunting for them I think* (P17/D)  *In the end it was like we can’t cope with this amount of numbers because you can’t do a wellbeing check if your numbers are too high because you’re rushing to get in and out too quickly…* (P17/D)  *Some people have not been out since the beginning of March. You can see how they’re mentally changing, how they’re getting depressed, how they’re frightened of leaving their own little flats or their houses now. If we get a second lockdown, I dread to think what’s going to happen* (P2/D)  *They don’t like you wearing the mask, because they want to see your face, so that hasn’t helped. It is also a little bit harder to communicate with a mask on if they are hard of hearing. So that has made it more difficult* (P1/D)  *We wear masks and that does make some of them a little bit happier. Then also others they’re going, “You’re not going to catch anything off me.” I go, “I know that, I’m wearing it for your protection.” It’s a lot more difficult* (P2/D)  *I’d say you’re more wary, because you’re wearing the mask it’s harder to communicate with them as well. You know what it’s like wearing a mask, when you’re talking they’re falling down. You’re very aware, aren’t you, how many times you touch that mask and you pull it up and you try and touch it the right way* (P2/D)  *And also when you're trying to speak to them as well. It was difficult. A lot of them were saying, "We can't hear you." It was scary. We just reassure them. "Well, this is for your protection and for mine. We have got to wear this for safety."* (P3/D)  *We’re wearing PPE so we’ve got masks on, and that makes an awful lot of difference, actually. It’s like putting a barrier between you and the client because, obviously, a lot of people are hard of hearing, so they find it quite difficult to talk to somebody in a mask. They can’t see your facial expression so well and I do like to go in with a bit of a cheery smile, and they can’t see that* (P4/D)  *If you have to go in the house, you’ve got to mask up, suit up, gloves on, wash your hands, and it all takes time. It all takes time, and, of course, every house you go in, when you come out, you’ve got to bag that kit up, and the next one, you’ve got to put another set back on* (P6/D)  *Doorstep deliveries, yes. Obviously, you know, the news can be quite intimidating at times if you listen to it, and probably quite fearful of the consequences of having strangers in their house. So we still doorstep a few deliveries because clients are worried about us entering their home. So, I would imagine that’s probably the easiest way round it* (P11/D)  *I would say that’s probably the biggest challenge, not being able to see their family, their friends, and being isolated and… Not even, maybe, being able to see their friends in that particular home because that’s where they’re vulnerable. Obviously, care homes got really hit* (P12/D)  *You use PPE and keep yourself as far away from them, if you can, as possible. But again, like I said, with people with dementia, they don’t really understand. Sometimes it could be quite difficult asking them to stay away, keep their distance from you because they find it quite upsetting* (P17/D)  *I think it's particularly hard on them. That even that small interaction is altered with the face mask and you've got to shout a bit more. It's just not as easy to have the sort of free flowing conversation that you would have had pre-COVID days. But I think mental health-wise, I think definitely they could easily struggle. I mean, on one level, their lives are quite lonely or isolated anyway* (P18/D)  *So one I saw today, he's basically trapped in his house. He has dementia, so it's not good for him to walk around by himself, obviously, in case he can't remember how to get back. But he is not seeing anyone and is on his own a lot and is quite scared of the outside world. So a lot of them, if their families aren't able to visit them, it's just quite a sad existence sometimes* (P15/D)  *I think the major effect is loneliness. It’s that isolation. Most of them live in quite a small world anyway. You know, they have either some, sort of, mental issues around dementia or they’re physically not as mobile as they used to be. So, very reliant on family or carers to do things for them. I think from that point of view, a great deal won’t change, but it may increase isolation because the frequencies may drop off of additional visitors to, sort of, if you like, services going in, because we’re not family* (P11/D)  *I would say that’s probably the biggest challenge, not being able to see their family, their friends, and being isolated and… Not even, maybe, being able to see their friends in that particular home because that’s where they’re vulnerable. Obviously, care homes got really hit* (P2/D)  *I think obviously yes, the family, loneliness and things like that probably becomes more apparent, but I think they resign themselves to it. It’s like, “Oh well, here we go again, it’s got to be done” and they do kind of just get on with it* (P14/O)  *I think it is really tough and I think it will be hard because there are some of them that hate just talking on the doorstep and they hate talking to me wearing a mask* (P18/D)  *Going on the first one, because we worked all the way through it, we didn’t stop, they were scared, very apprehensive, didn’t understand what was going on. Those are the three things that stand out. They knew something was happening but they didn’t really know. They haven’t got the capacity, some of them, to understand what is going on. They see you turning up with a mask and, “What have you got that on for? Why are you doing that?”* (P1/D)  *When you’re arriving with your mask on and you can’t always go in there, it’s hard for them, definitely. They get quite offended as well because you’re in there wearing the mask. They think you’re going to catch something off of them. It’s reminding them, “No, there’s this horrible virus about.” It’s hard for them as well* (P2/D)  *I find when I go in there, even though I’ve got the mask on, I’m still doing my 2m away from them. I find it has changed how you do interact with them. You need to get out as quickly as you can because you don’t want to put them at risk any more than you’ve got to* (P2/D)  *Especially when they saw us with all the PPE as well, that was a bit scary for them* (P3/D)  *Well, that’s changed a lot, you see, because of COVID. We used to be able to dish the food up onto plates. So, we would go in, get them cutlery, get them a glass of water, maybe, put the food on a plate, put it in front of the client. But we can’t do that anymore. So, it is a real shame* (P4/D)  *I think the effect that it will have on some clients will be quite bad, actually, because I’ve noticed that, particularly clients who lives in the flats- Well, no, that’s not really fair. A lot of the clients have quite a busy social life. They go to coffee mornings and that sort of thing, and church and groups and lunch groups and all that, which, of course, all had to stop. Mood has changed quite a lot, really. Some people are quite depressed at not being able to see and go out and do these things* (P4/D)  *I think if a second wave came, it would be awful for them because they’re just beginning to feel that- You know, one of my ladies today, she said, “Oh, I managed to go to church on Sunday. It’s so lovely to see everybody.” They’re just beginning to be able to go out again and go to the pub for a meal, which some of them do with their families, and go into their families’ gardens at weekends, and that sort of thing. It’s all beginning to happen again and I think if we went into lockdown again it would be quite bad, really. It’d be hard for them* (P4/D)  *Yes, I think the service may have changed in so far as that more people are having doorstep deliveries and continuing to do that. They're still not confident. They're still not confident about us coming in and just… But enjoying the doorstep delivery, nevertheless* (P5/O)  *Now we go. There were quite a lot of doorstep deliveries because of COVID, but I think, obviously, in the longer run particularly with some of the, sort of, concerns over dementia, it’s probably better if we can, with their consent, go in and serve it to them. So, I think there have been three changes on my round during the last three months where we’ve gone from doorstep delivery to actually going in to plate, just because we were concerned about the fact that they weren’t eating the food* (P11/D)  *So, I think from that point of view, a great deal won’t change, but it may increase isolation because the frequencies may drop off of additional visitors to, sort of, if you like, services going in, because we’re not family. You know, we go in to provide a service, the same as the carers do, and although it can be quite personal, it’s not the same as having your own family around or friends. So, yes, I think that would be the biggest effect on most of them* (P11/D)  *There was a couple that had possible symptoms, so we were told, so that would be a doorstep delivery. They would take the meal, they would put it in a box, put it by the door, ring the door. Wait for the person to come out, because there has still got to be a wellbeing check even if they’re not going in the house. At least they have to ensure that they saw the client to make sure that they were okay. Then once they had seen the client and they were happy; they would come away and the client would take the meal in* (P14/O)  *I suppose some people were scared, so people who might go into the house and put the food on the table or something were taking it on the doorstep if they could. Or I had one customer who made me put it on the doorstep and then walk away to minimise being near anyone* (P15/D)  *But occasionally, they can go into a customer and just say that customer, with COVID-19, doesn’t want us to go into the house. So, it’s more or less a doorstep delivery. So, you would only be there a minute* (P16/O)  *For the client's sake, the difference is that more are doorstep deliveries. Whereas ideally, you would go into most people's homes, so you can get a feel for how they are and how they're living. You notice more things. Whereas, with COVID, if they can take the meal at the door now, either on a tray or bring the trolley or whatever, then we try and do doorstep deliveries. It doesn't work all the time. I still go into probably just under half, I still deliver actually into their homes because it just doesn't work for everybody. They have said, "I'm fed up with this. I can't wait until we're back to normal. Until I can go back into the homes." It is their choice that I'm delivering, to a degree, at the doorstep. Although, I suppose, we were saying that's the way we wanted it to go for their safety* (P18/D)  *When we started we gave the service users the choice. “Leave us a little bench out or something and we will put it on there. Or, if you are happy for us to come in, we will carry on bringing it in.” We have got the PPE on. We have got our masks on. We have got everything else like that on. On my round, out of the twenty-eight, I only had two that wanted me to leave it outside. Everybody else I have gone in* (P1/D)  *There is another round where the driver is leaving every single meal outside. I think that is dangerous, because when the person tries to pick it up, because of your age and your balance, you are going to topple over. If there is any way that I can get in a house I will get in a house. I don’t like leaving it outside* (P1/D)  *Yes. I think the council’s stance on it is they want us to do doorstep, when you can. I disagree with that, because I think if it is safe to go in there, and they are happy for you to go in there, and the family are happy for you to go in there, you should go in there. That is my take on it. Other drivers have different opinions on it. As I say, one round the driver doesn’t go into hardly any of theirs. I cannot see how you can do a welfare check. I think I actually said to my manager, “You might as well get Asda to deliver.”* (P18/D)  *‘Well, if the person comes to the door they will just see them. I imagine they will just say, “Are you okay?” and they will say, “Yes.” But you can’t gauge anything from that’* (P1/D)  *And you can’t check that they are eating their meals. Because that is another thing I do. When I go in there I make sure they have eaten their dinner from the day before. Because if they are not they will put the hot dinner in the fridge. So I always check the fridges, make sure they haven’t put it in there from the day before. We take teas out as well, which is sandwiches, a bit of cake or a trifle. You have to check on that, to make sure they are eating those, because sometimes they will put that in the fridge. They will have two or three days’ worth of food in there. So you have to throw that food away and make sure that they are eating it. You cannot do a welfare check from the front door* (P1/D)  *Just to make sure that we do the welfare check. I can’t emphasise how important that is. If drivers are not prepared to go in someone’s house, even though it has obviously got to be safe, if they are not prepared to go in there then don’t come and do the job. You have still got to get out there. You have still got to go in that person’s house. You have still got to see everything they are doing. That is why I say doorstep doesn’t work. I am of the mind that if you are anxious and you don’t want to go inside someone’s house then you should be taken out of the job while the lockdown is in progress. Get somebody who is going to go in there and do the job. If you are anxious, that is just one of those things, isn’t it? That is just how you are. But it is not helpful* (P1/D)  *If you can do a doorstep delivery, then yes. I worry because some of the drivers put them on the floor. I worry if they bend down to get the dinners they’re going to fall over* (P2/D)  *I find it very hard when you deliver it in the door because it’s hard to do that welfare check* (P2/D)  *That would worry me because you’re not seeing what’s going on inside that house. You won’t notice things. If you’re delivering at the doorstep, you’re not having that same chat that you’d have because you’re going off. That would worry me* (P2/D)  *Possibly loneliness would be the biggest one, I would say, that would be the biggest concern of the second wave of this, if customers have been self-isolating all that time and now, they’ve got to go on even longer. It’s bound to, I would say, have a bit of an impact on them, because it’s going a long time, isn’t it?* (P16/O)  *That even that small interaction is altered with the face mask and you've got to shout a bit more. It's just not as easy to have the sort of free flowing conversation that you would have had pre-COVID days* (P18/D)  *The protective equipment that I wear can be time consuming to put on and seems impersonal but is to protect myself and the service users… I would have to go to wearing aprons, which, they are time consuming because you’ve got to – they’re like trying to open up a polythene bag, you know, where they’re stuck together. You’re trying to open them up and tie them up and then you’re going to be serving less customers* (P10/D)  *Some people you would have to do doorstep delivery, but if it's somebody who can't physically manage themselves, like those who we have to plate up for, you would have to go in. You would have to go in because otherwise, they wouldn't get a meal. They need that extra help* (P3/D)  *You cannot do a welfare check from the front door…* (P1/D)  *So yes, there was quite a lot of maybe paranoia and anxiety. And obviously it's still going on, but it wasn't particularly nice to work in* (P15/D)  *… maybe just anxiety levels, I suppose, because it feels like a responsibility to not make people sick* (P15/D)  *We said that just before the first lockdown. It was like, “How does this work? Have we still got to work?” They were like, “Yes, you’ve still got to come in,” which was a bit frightening at first because like I said, no one really understood it* (P17/D)  *The amount of anxiety within the drivers is completely understandable* (P5/O)  *Yes, and then also, if there are particular staff members who are finding it difficult, we come up with stress and well-being plans. We call them health and well-being plans, where we come up with a road map of are there any adjustments we need to do to support them, and work, if they need to take time off we are quite flexible with that. Even finding cover, we are quite well-resourced. So we are able to manage that. If we think it is affecting someone in particular, we can offer various support mechanisms to help them* (P13/O)  *No one was being tested. We didn’t have any PPE to start with. It was quite frightening, especially when you’ve got children at home and you’re out all day, then you’re coming home* (P17/D)  *It’s a worry for the drivers going into properties. Because you can’t socially-distance when you're with elderly people who have dementia. How can you explain to someone who has got dementia, “You have to stay away, you can’t come near me”?* (P7/O)  *Obviously at the beginning it was stressful because we didn't know what was going on. We were so worried about catching COVID and not knowing and then spreading it* (P15/D)  *We have tried to make sure that we have made everybody as [Covid] aware as possible and had the correct equipment to do their job as correctly as possible… Then obviously to offer the employee assistance helpline… to come and speak to us because then we can help with that, we can help change things to support them* (P13/O)  **- Concerns about the future of MoWs, particularly in further lockdowns**  *Obviously, if you’ve got a client who can’t move, who is bedridden or we use key safes or whatever for, then they would just mask, aprons, gloves and just go in and kind of stay away. You know, stay as far away from the client as possible, but they would go in* (P14/O)  *We have gone through our customer base and we have seen who we consider to be very vulnerable, least vulnerable…we do have contingency plans in place. We have a large pool of human resources we can request for support from* (P13/O)  *I can't see it changing. I imagine we would carry on. I think because of the job we do, it will just carry on and possibly get busier* (P18/D)  *It just carried on, with just extra precautions…I think we’re better prepared if it happens again* (P14/O)  *I think we’re better prepared if it happens again. So our stores person worked to see how we could arrange the freezer. So that we could cut down the delivery days but have more meals in, so that we always have a good stock* (P14/O)  *I think there will be a lot more demand, a lot more pressure* (P7/O)  *I would expect some people, where they’ve been coping so far, may find that an additional stress of more restrictions may promote them to look for more support and we may be one of those services* (P11/D)  *I think the only biggest issue for me would be down to staffing* (P13/O)  *Because of family or because they’ve got symptoms. I mean that would put a huge pressure on the service. I mean hopefully yes, [manager] would be able to call on some staff from the day centres again, to come and help. We are now in the process of recruiting at the moment, but they would have only just started, so it would be a bit hard to dump them in at the deep end* (P14/O)  *Our main concern is if one of us goes down with it then we would all have to self-isolate. Then that would become a big problem* (P17/D)  *The kitchen area, that was a change. Fortunately, for most of it, the weather was good, so we could distance outside. It will be testing if it carries on hugely into the winter, about where we can be geographically in the building. I meant when we are at the kitchen, waiting to collect the meals. At the moment, we all wait outside and only go in when the meals are ready. But if it's pouring down with rain or freezing cold, then more of the drivers are going to have to be inside. So, we will have to make sure we isolate, distance indoors* (P18/D)  *I think it is really tough and I think it will be hard because there are some of them that hate just talking on the doorstep and they hate talking to me wearing a mask* (P18/D)  *But we do know that we need to support people more, definitely. Especially if people need to be isolating. We need to be able to get more meals out to people, definitely. More checks on people* (P3/D)  *The people are staying at home and they just… we have quite a few new clients where the relatives got us in to do it for them. So, if there’s a second lockdown, I expect the very same thing is going to happen* (P6/D)  *… before lockdown the drivers used to do their booking in with the Customer Service Advisers, they used to walk into the office, and stand by the side of you, and talk through their round sheets, what they’d done that day, and anything that needed to be changed or altered on the Service for that customer, now, we are – nobody comes in the office unless they’re invited now. It does take longer to see the drivers in but, I mean, it’s just something that has got to be done, isn’t it? I must admit, when it’s raining, then we let the drivers come round the other way into the kitchen. So, they’re waiting in the kitchen area* (P9/O)  *I think it will be exactly the same as in March. If we lockdown again, we would just continue the same way as we always have* (P10/D)  *I don’t see there being any change. During the first wave, I joined. I was quite surprised to get the job because we were still really not… we hadn’t come out of relaxing of the lockdown conditions when I got the job. So, I started the job during lockdown. So, I don’t see the role changing. We will continue to do what we do. You know, it’s emphasised to us, we carry PPE in all the vans, I wear a mask, we sanitise after every delivery through hand gel, and the client-base really, by and large, are living independently in their own homes. So, in terms of risk assessments and any changes to that, they’ve already been carried out. So, we continue as normal, I would hope* (P11/D)  *No. You know, my line manager is very conscious of not putting on so many deliveries that we feel under pressure ourselves, and equally, that we deteriorate in terms of the attention that we give to the people we deliver to. So, I think the attitude would be that they would seek… like, we’ve got people from other services currently supporting us and it was discussed only this morning with the manager, that if we as a group have a COVID outbreak, they’ve got contingency plans to bring additional drivers in from other services that may not be as busy within the city council* (P11/D)  *But we do have contingency plans in place. We have a large pool of human resources we can request for support from. We just need to make sure that we have a training package that is almost like a pick up and go in the instance that someone does come on very last minute, which we experienced last time, but because we have had the staff redeployed, they are now going back to their original service areas. They are our first point of call. They are already trained on the programme, so we can call upon- They would be our first line, should I say, of calling back to come and support us here if staff took off* (P13/O)  *I think so. I think that it dealt with it quite well the first time and, you know, that was just a very unusual circumstance. So they've kind of done it before, so maybe, I reckon it's... Yeah, I think it will be prepared* (P15/D)  *But, I mean, we’ve gone through it quite well. I mean, the staff were very anxious when it first came out. They are not so anxious this time around, I think, because they’ve been working through it and they- I don’t know, they, sort of, understand the use of PPE more regular and everything, and they’ve got used to it. I think it has become part of their everyday routine. So, the drivers are not so anxious this time around* (P16/O)  *Hopefully they will be used to it because of the first lockdown. Like with everybody, no one really knew what was going on with the first lockdown or what was acceptable, what wasn’t acceptable. My personal view is I don’t think it would affect them that much because we’re still going in with masks on, PPE for dishing up meals and things so I don’t think it would- I still try to get as many clients to come to the door as I possibly can because then it stops me having to go in to too many houses and putting more people at risk. As I say, most of them are pretty good now. They are quite trained and they do come to the door with a towel or a tray. It’s not too bad at all* (P17/D)  *Now I wouldn’t have any worries really about doing it in a second lockdown because we managed quite well the first time. Like I say, it was quite full on but we managed okay so that was fine* (P17/D)  *To be honest, I haven’t changed that much. As long as it is safe and I have kept my distance I will still do all the other things that I have been doing all the other time. Take the rubbish out, change the lightbulbs etc. It doesn’t have a bearing on that because I am still going in the house* (P1/D)  *If that is the way they are going to go. Because we have something that is called casual drivers. They come in. If we have a holiday, they will cover us for our holiday. If someone goes sick, they will cover that person for sick. They are not contracted but they know how to do the job. So you have casuals and they could come in* (P1/D)  *I don’t think it would change so much this time, because they are used to us wearing masks and stuff now. So I think the second spike would be easier. Not just for them but for the drivers as well, because we have got a handle on it now, what to do. But it was quite surreal. It was quite surreal* (P1/D)  *I don’t think it will change anything with meals. We got really busy at the start of it because obviously families were putting more on so we could keep an eye on them. I don’t think it will affect the meals on wheels service. With the meals service, I don’t think it will affect us because we’re still working as best we can and doing as best we can that we’ve always done* (P2/D)  *Yes, I do, actually. I do, I think we’ve been very well prepared. We’ve had lots of PPE. We’ve got no shortage of that at all and I think we’ve all coped pretty well, actually* (P4/D)  *We’ve got a nice big stock of gloves. We're a bit short of masks, but I do keep a little stock because, again, that's what I've been looking after, the PPE. We then go to our Business Support team and ask them to order some more. We don't have enough at this moment, I don't think, because, if it really does kick off again, we might be doing some doorstep delivery. We're addressing that at the minute, with the masks* (P5/O)  *Like I said, we do have a couple of extra drivers from different departments, but because of the way we work, the office staff, they would come in and help us. It’s not us and them. We all are trying to achieve the same goal. It’s not like because you're sat in the office behind a computer, you can’t come out, get in a van and go and deliver 20 meals. I mean, they all do* (P6/D)  *Yes, we can handle it anyway. We handled it last time. We can do it this time. We’ll deliver meals regardless. Yes, they know they’re going to get a hot meal, regardless of anything* (P6/D)  *We know now what we’re in, don’t we, we’ve done it once? So, I think everyone is fully aware. We still worked throughout lockdown and throughout the pandemic, our work doesn’t stop. So, yes, it is quite pressured. But we’re all trying to do everything that we can, to the best we can, really… when I walked in there, everything they put into place straightaway was just amazing. So, I think, hopefully, the drivers would say the same, I hope they feel that they're protected and they're as safe as they can be. Yes, we’ve certainly done everything we can* (P7/O)  *I think they're in the middle of recruiting at the moment, I think interviews are taking place next week for two positions. So, yes, hopefully, if those come on board and they can train them, etc., that should ease the pressure a little bit* (P7/O)  *As far as the clients are concerned with us, probably not a lot will change. Because all our drivers go in with PPE on anyway and they’re still being looked after and they’re still having their meals. So, I can’t see, as though, from a client’s point of view, much would change there, anyway* (P9/O)  *Management deal with that but, yes. We would be able to have other drivers come and help us out and even if we had to have different vehicles from fleet services, we would be able to cook the meals in the kitchen and give them a few meals in a hot lock to deliver. So, yes, they would still go out hot, even if they didn’t have the vans and it cooked the meals in the back of the van* (P9/O)  *So, if it happens again, it happens again. I mean, we’re just all on standby, basically. We all are, yes, yes. We all try to do our bit, yes. It’s not just teamwork, it’s almost like family at work, you know? It’s just all muck in where you can* (P9/O)  *… maybe our numbers will go up again. I think we might have people come back on who have since stopped having them, since last time. I should think that might happen, actually, because, probably, word will have got around that it’s quite a good way of coping* (P4/D)  *Yes, it’s been really important to know where we’re getting all those [meals] sourced from and, if that was to fail, what happens next* (P12/D)  *It was quite stressful but I do feel, me personally, if we did go into a second lockdown then we know a lot more about what we’re dealing with than we did the first time round* (P17/D)  *I think that is the whole crux of the matter.We have got to keep doing what we are doing* (P1/D)  *Well, we have to carry on. I mean, it would be needed even more… because we did have a lot of extra people on during that time we were isolating* (P3/D)  *We've taken on casuals. We've got two people covering rounds so, hopefully, we'll be in a good position. We’ve got more staff, definitely. It seems to be better organised, yes. Yes, I think I do feel in a better place than we did, we were originally, yes, definitely* (P5/O)  *We know food wise now what we can do and what we can hold in the freezers, and stuff like that, so we can plan more in advance if we know that there is going to be an issue with deliveries. I think it’s just all round preparation, and then we just carry on as normal* (P14/O)  *I think we are quite well-rehearsed now using PPE. That is not something to be scared of, that we are scared of anymore. We are well-stocked with PPE* (P13/O)  *So services such as the meals service are going to become even more important because we simply, I feel, don't have the infrastructure to meet the demand of what to expect over the next few years, particularly as there have been so many cutbacks over the past few years* (P13/O)  *If there was a second spike and more people came on, ideally… you would use your casuals daily and you could split a round of 30 down into two rounds of 15, which would give you more time with each one, which would be a better way of doing it. But again that is up to the council. Would they want to pay two people to do one person’s job? I don't know* (P1/D)  *Yes, absolutely and it's short-sighted to think that it's something that you could perhaps cut back on and expect people still to be able to manage* (P18/D) |
